# Multiomics Analysis Identifies Chromosomal Instability‐Associated Immune‐Related Signatures in Hepatocellular Carcinoma by Integrating Weighted Gene Coexpression Network Analysis (WGCNA) and Machine Learning

**DOI:** 10.1155/humu/6594696

**Published:** 2026-04-19

**Authors:** Zehao Li, Boqiang Zhong, Qian Zhang, Lin Sun, Xiaoxiao Li, Xiao Hu

**Affiliations:** ^1^ Department of Pancreatic Disease Treatment Center, The Affiliated Hospital of Qingdao University, Qingdao, Shandong, China, qdu.edu.cn; ^2^ Medical Affairs Department, The Affiliated Hospital of Qingdao University, Qingdao, Shandong, China, qdu.edu.cn; ^3^ Department of ICU, The Affiliated Hospital of Qingdao University, Qingdao, Shandong, China, qdu.edu.cn; ^4^ Center for GI Cancer Diagnosis and Treatment, The Affiliated Hospital of Qingdao University, Qingdao, Shandong, China, qdu.edu.cn

**Keywords:** chemotherapy sensitivity, hepatocellular carcinoma (HCC), immune microenvironment, machine learning, risk scoring

## Abstract

**Background:**

Hepatocellular carcinoma (HCC) is a top cause of cancer‐related death globally, with late diagnosis due to nonspecific early symptoms. Current single‐factor prognostic models cannot reflect tumor heterogeneity, so a comprehensive tool for risk stratification and personalized treatment is needed.

**Methods:**

This study employed WGCNA on publicly available datasets (TCGA and GSE54236) to identify core genes associated with chromosomal instability (CIN) in HCC. We initially screened 73 candidate genes, which were then refined to a final set of 20 core genes through an optimization process involving 101 machine learning algorithms. Specifically, the StepCox[both] combined with CoxBoost model was selected as the optimal model, with a concordance index (c‐index) of 0.709. We subsequently developed a multidimensional risk‐scoring model by integrating the expression levels of these core genes with patient clinicopathological parameters and immune cell infiltration data. The model′s performance was evaluated through survival analysis and chemotherapeutic drug sensitivity prediction. Additionally, functional assays were conducted to validate the roles of key genes in promoting the proliferation and invasion of HCC cells.

**Results:**

The model effectively stratified patients into high‐ and low‐risk groups. High‐risk patients exhibited poorer survival, increased immune cell (particularly T cell) infiltration, higher sensitivity to chemotherapeutics like 5‐fluorouracil and paclitaxel, and a higher TP53 mutation rate. Low‐risk patients were characterized by frequent CTNNB1‐ARID2 comutations and a more active antitumor immune microenvironment. Additionally, SSRP1 and SETDB1 were verified to promote the proliferation and invasion of HCC cells.

**Conclusion:**

This integrated model, combining genomic and immunological features, is a reliable prognostic tool for HCC patient stratification and personalized chemotherapy, promising for clinical translation and precision medicine in HCC.

## 1. Introduction

Hepatocellular carcinoma (HCC) is a formidable global health challenge, ranking as the third leading cause of cancer‐related mortality [1, 2]. Although recent advancements in diagnosis and treatment have been made, the disease continues to present significant prognostic difficulties due to its high invasiveness, frequent recurrence, and the complexity of managing advanced stages [3]. The inherent molecular and clinical heterogeneity of HCC further complicates effective diagnosis and treatment, highlighting the urgent need for personalized therapeutic strategies.

Systemic treatments for advanced HCC, including chemotherapy and immunotherapy, face challenges from patient‐specific factors such as drug resistance, adverse effects, and limited response rates [4]. Although Sorafenib has long been the standard of care, its efficacy is often hampered by the development of resistance. Similarly, although immune checkpoint inhibitors (ICIs) like nivolumab have emerged as promising options, their overall low response rates and the lack of reliable biomarkers for patient selection underscore a critical gap in clinical decision‐making. A major contributing factor to this uncertainty is the highly immunosuppressive tumor microenvironment characteristic of HCC [5].

Chromosomal instability (CIN), a fundamental hallmark of cancer, is defined by a high rate of chromosome number or structural abnormalities. At a molecular level, CIN drives continuous genomic mutations, fostering tumor heterogeneity and playing a pivotal role in the progression of various malignancies, including HCC. In HCC, aberrant CIN activation has been directly linked to disease progression, treatment resistance, and poor prognosis. Given its central role in HCC pathogenesis, CIN represents a crucial focal point for both understanding the disease and developing precision therapeutic strategies [6, 7]. Despite the development of various prognostic indicators and predictive models in HCC research, significant limitations remain in their clinical translation. Many models fail to adequately account for the high degree of tumor heterogeneity, whereas others rely on single‐dimensional indicators, limiting their predictive accuracy and applicability [8]. To address these challenges, this study leverages CIN as a core pathogenic mechanism.

Using weighted gene coexpression network analysis (WGCNA), we systematically identified key genes coexpressed with 32 previously established CIN‐related core genes [9], using both The Cancer Genome Atlas (TCGA) dataset and an independent validation cohort (GSE54236). By integrating these genes with clinicopathological parameters, we developed a novel RiskScore model. This multidimensional model effectively stratifies HCC patients based on their overall survival and provides quantitative risk assessment. Furthermore, our analysis reveals significant associations between CIN‐related genes and immune cell infiltration, offering new insights into the immune regulatory mechanisms of HCC. The model also predicts drug sensitivity, enabling personalized chemotherapy and immunotherapy plans. Our RiskScore model provides a comprehensive framework that integrates genetic, clinical, and immune data, presenting a dependable tool for risk assessment and optimizing therapeutic strategies in HCC, thereby advancing the field toward a precision medicine paradigm.

## 2. Materials and Methods

### 2.1. Data Collection and Preprocessing

Gene expression profiles and related clinical data for HCC were primarily obtained from TCGA data portal, which includes 374 HCC samples accompanied by their respective clinical information. In addition, the GSE54236 HCC dataset, consisting of gene expression profiles and clinical data from 80 HCC samples, was obtained from the GEO database. To maintain consistency and improve comparability between these datasets, batch effects were adjusted using the ComBat function implemented in the sva R package.

### 2.2. Feature Selection and Risk Score Model Construction

A set of CIN‐related genes was established, and CIN scores for each HCC sample in the TCGA dataset were calculated using GSVA. WGCNA was then applied to identify the top 200 genes most correlated with CIN. Univariate Cox regression was performed on these genes in both TCGA and GSE54236 datasets to select prognostic genes (*p* < 0.05). The overlapping prognostic genes from both datasets formed the final risk gene set. Using their expression data, 101 machine learning models were developed to predict patient risk and prognosis.

### 2.3. Survival Analysis and Model Validation

To thoroughly evaluate the prognostic reliability of the HCC risk model based on CIN‐related genes, a comprehensive validation approach was used. First, time‐dependent ROC curves comparing the model and clinical factors (e.g., age and tumor stage) were generated with the “timeROC” R package to assess AUC at 1, 3, and 5 years. Next, the concordance index (C‐index) was calculated using the “rms” and “pec” R packages with bootstrap cross‐validation (*B* = 1000) to reduce bias and measure overall prediction accuracy. Subsequently, univariate and multivariate Cox proportional hazards regression analyses were conducted utilizing the “survival” package to evaluate the independent prognostic significance of the risk score while controlling for tumor stage and additional clinical covariates. Hazard ratios (HRs) accompanied by 95% confidence intervals were reported. Thereafter, patients were stratified into high‐ and low‐risk cohorts according to the median risk score, and the prognostic discrimination of the model was assessed through Kaplan–Meier survival analysis alongside log‐rank testing, with statistical significance set at *p* < 0.05.

### 2.4. Functional Enrichment Analysis

Differential gene expression was assessed using the “limma” R package. Gene Set Enrichment Analysis (GSEA) was performed with “clusterProfiler” (v4.12.0) to identify significantly enriched KEGG, GO, and Hallmark pathways, applying a false discovery rate (FDR) threshold of < 0.05. For immune analysis, single‐sample Gene Set Enrichment Analysis (ssGSEA) quantified immune pathway activity per sample, and immune cell infiltration across 28 subtypes was estimated using deconvolution methods such as CIBERSORT. Spearman correlation tested associations between the risk score and immune infiltration, considering *p* < 0.05 as statistically significant.

### 2.5. Immune Cell Infiltration Assessment

This research leveraged a comprehensive toolkit of immune infiltration analysis platforms—namely Quantiseq, TIMER, MCP‐counter, xCell, CIBERSORT, and EPIC—to map out the landscape of immune cell populations within tumor tissues. The “estimate” R package was employed to get a handle on tumor purity, stromal components, and immune cell presence, painting a clearer picture of the tumor microenvironment. On top of that, the Tumor Immune Dysfunction and Exclusion (TIDE) method was brought into the fold to assess how tumors might be dodging the immune system. By tying everything back to the risk score, the study put high‐ and low‐risk groups under the microscope when it came to predicted immunotherapy responses, hinting that this risk model could be a game‐changer for forecasting treatment outcomes.

### 2.6. Genetic Variation Characteristics

To elucidate the genetic variation patterns associated with the CIN‐related HCC risk model, a multidimensional analytical approach was undertaken. First, somatic mutation annotation and characterization of the TCGA‐LIHC dataset were performed using the “maftools” R package. Single nucleotide variant (SNV) waterfall plots were generated to visually depict intersample heterogeneity in gene mutation profiles, with special focus on the mutation distribution of CIN‐related genes such as TP53. Second, gene comutation heatmaps were constructed to identify patterns of co‐occurrence and mutual exclusivity among frequently mutated genes, including TP53. Statistically significant comutation events were determined using Fisher′s exact test with a significance threshold of *p* < 0.05, thereby revealing potential genetic interactions and their biological implications.

### 2.7. Drug Sensitivity Prediction

This research crafted a novel pharmacological response projection system leveraging genomic information to pinpoint precision therapeutics for individuals battling liver cancer. By integrating medication efficacy metrics from specialized bioinformatics tools—the “oncopredict” and “pRRophetic” R packages—researchers evaluated treatment susceptibilities across patient samples. Using Spearman′s correlation method, they examined connections between the calculated risk assessment and drug concentration thresholds required for 50% inhibition. Chemotherapeutic agents demonstrating statistically meaningful associations (*p* < 0.001) were singled out, thereby strengthening the foundation for customized therapeutic approaches in HCC management.

### 2.8. Statistical Analysis

All statistical analyses were executed using R software, with statistical significance established at a two‐sided *p* value threshold of less than 0.05. Variables conforming to a normal distribution were assessed using Pearson′s correlation coefficients, whereas Spearman′s rank‐order correlation was adopted for variables violating normality assumptions. Between‐group comparisons involving two independent samples were conducted via independent samples *t*‐tests for parametric data and Mann–Whitney *U* tests for nonparametric datasets. For comparisons across multiple groups, one‐way analysis of variance (ANOVA) was employed for normally distributed variables, whereas the Kruskal–Wallis test was applied for nonparametric data. Survival differences between cohorts stratified by CIN risk scores were evaluated using Kaplan–Meier survival curves coupled with log‐rank tests.

### 2.9. Reagents and Antibodies

Small interfering RNAs (siRNAs) targeting SSRP1 and SETDB1 were synthesized and constructed by Genepharma Chemical Technology Co., Ltd., Suzhou, China. All antibodies used in this study were purchased from Proteintech Group, Wuhan, China, including anti‐SSRP1 antibody (Cat. No. 15696‐1‐AP), anti‐SETDB1 antibody (Cat. No. 11231‐1‐AP), and anti‐GAPDH antibody (Cat. No. 10494‐1‐AP).

### 2.10. Cell Culture and Transfection

Normal liver MIHA cells and HCC cell lines, including Hep3B (3B), PLC/PRF/5 (PLC), Huh7 (H7), and MHCC97H (97H), were obtained from the American Type Culture Collection (ATCC). All cell cultures were maintained in a humidified incubator at 37°C with 5% CO_2_. Cellular morphology and proliferation rates were monitored every 8–24 h via inverted microscopy. Subcultivation was carried out upon reaching 80%–95% confluence by enzymatic detachment using 0.25% trypsin‐EDTA, followed by centrifugation at 1000 rpm for 5 min and resuspension in fresh growth medium. For siRNA transfection, cells were seeded into 6‐well plates and transfected at 70%–80% confluence employing Lipofectamine 3000 reagent, with siRNA diluted in Opti‐MEM according to the manufacturer′s instructions. The transfection medium was replaced after 6–8 h, and cells were harvested 48 h posttransfection for subsequent functional assays.

### 2.11. Western Blot (WB) Analysis

Adherent cells were lysed on ice for half an hour in cold RIPA buffer supplemented with protease and phosphatase inhibitors. The lysates were then centrifuged at 12,000 × g for 15 min at 4°C, and the resulting supernatants were collected for protein quantification using the BCA assay. Equivalent amounts of protein were combined with 5× SDS‐PAGE loading buffer and heated at 100°C for 5 min to denature prior to electrophoresis.

A 5% stacking gel (pH 6.8) alongside a 10% resolving gel (pH 8.8) was cast for SDS‐PAGE. Electrophoretic separation was conducted initially at 80 V for stacking, followed by 120 V until the dye front reached the bottom of the gel. Proteins were then transferred onto PVDF membranes activated with methanol using a Bio‐Rad wet transfer apparatus at 300 mA for 1 h.

Membranes were blocked with 5% nonfat dry milk in TBST at room temperature for 2 h, then incubated overnight at 4°C with primary antibodies diluted in the blocking solution. After washing steps, membranes were exposed to HRP‐conjugated secondary antibodies for 1 h at room temperature. Protein bands were visualized using an ECL detection reagent and imaged by a chemiluminescence imaging system. GAPDH was used as an internal loading control.

### 2.12. EdU‐488 Cell Proliferation Assay and Transwell Invasion Assay

Log‐phase cells were seeded into 6‐well plates and cultured until 30%–40% confluence. Subsequently, cells were incubated with 50 *μ*M EdU for 2 h at 37°C. After washing with PBS, cells were fixed in 4% paraformaldehyde and permeabilized with 0.3% Triton X‐100. EdU incorporation was detected by incubation with Alexa Fluor 488 Click reaction solution in the dark, followed by Hoechst counterstaining. Fluorescent images were captured randomly from five fields per sample.

For invasion assays, Transwell inserts with 8 *μ*m pores were precoated with diluted Matrigel and polymerized at 37°C. Log‐phase cells were suspended in serum‐free medium and seeded into the upper chamber, whereas the lower chamber contained medium with 20% FBS as a chemoattractant. After 48 h incubation at 37°C with 5% CO_2_, invaded cells on the lower membrane surface were fixed, stained with crystal violet, and counted in five random microscopic fields following the removal of noninvading cells.

## 3. Results

### 3.1. Identification of HCC‐Related Genes Through WGCNA Analysis of 32 CIN‐Associated Genes

Based on the 32 CIN genes identified in the study by Drews RM et al. (Table S2), we performed WGCNA to screen CIN‐related genes in the TCGA and GSE54236 datasets. The analysis revealed that the magenta and blue modules exhibited the strongest association with CIN (Figure 1a,b). From these two modules, the Top 200 genes with the highest correlation to CIN were selected (Table S4), and the Top 50 most strongly correlated genes are highlighted in Figure 1c,d.

Figure 1(a, b) WGCNA module‐chromosomal instability (CIN) association heatmaps of TCGA and GSE54236 datasets. (c, d) Hazard ratio heatmaps of Top 50 CIN‐related genes in magenta/blue modules of two datasets. (e) Venn diagram of the intersection of CIN‐related prognostic genes in two datasets, with 73 candidate genes obtained. (f) Comparison of average C‐index of 101 machine learning models. (g) Circos plot of chromosomal localization of 20 core genes screened by the optimal model; arcs of different colors represent chromosomes, nodes are gene locations, and lines indicate gene coexpression associations.

**Figure:**
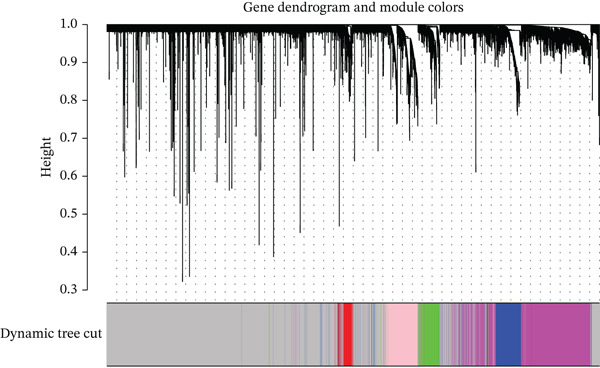
(a)

**Figure:**
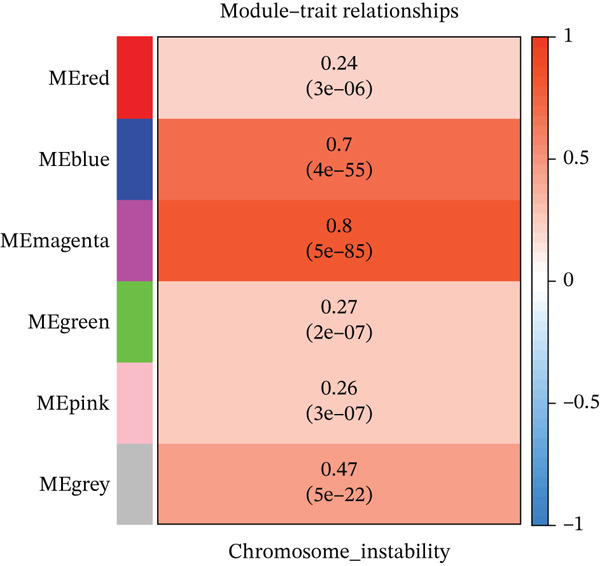
(b)

**Figure:**
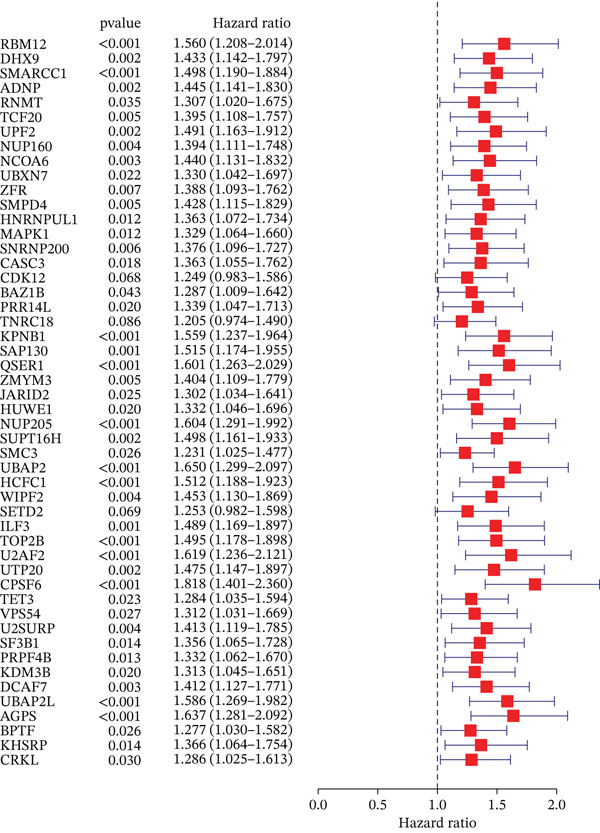
(c)

**Figure:**
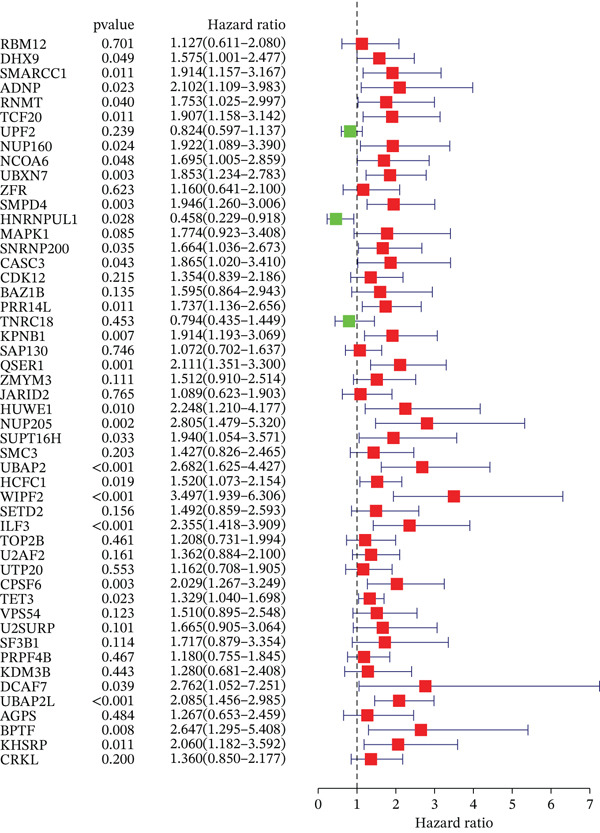
(d)

**Figure:**
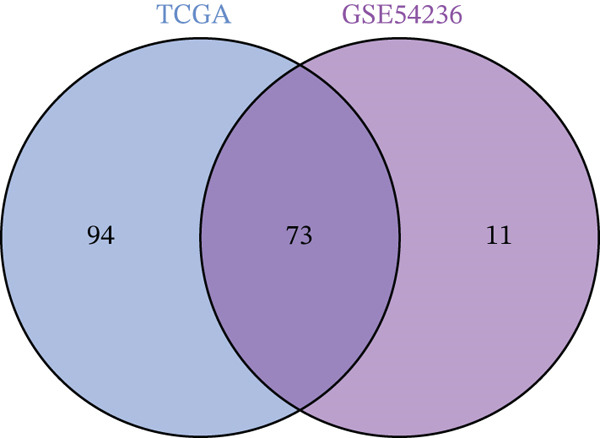
(e)

**Figure:**
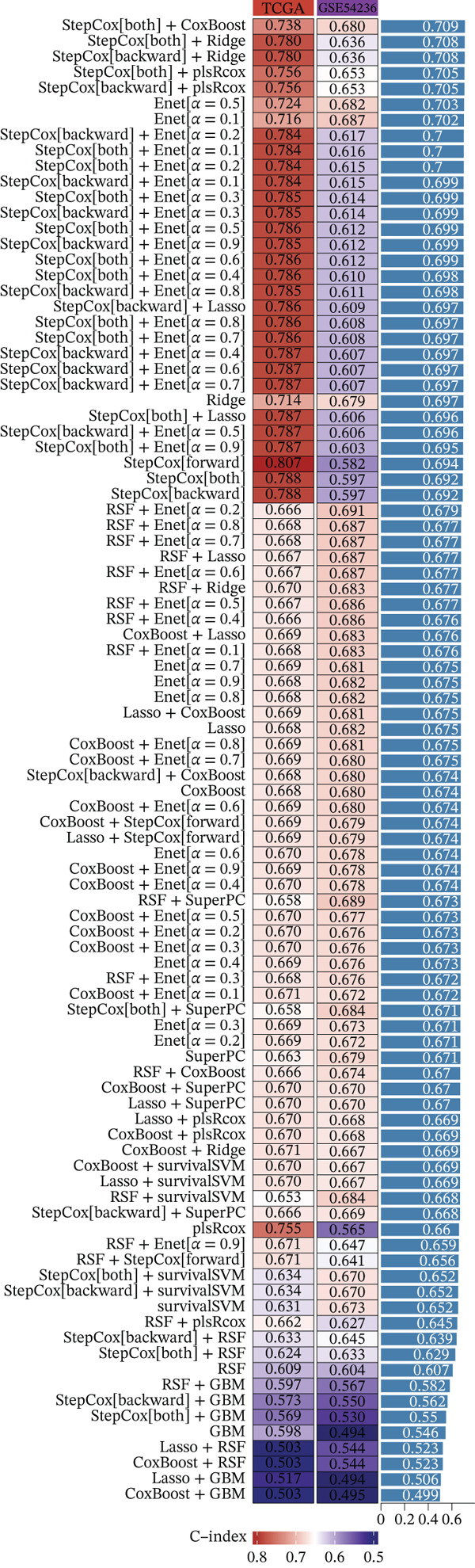
(f)

**Figure:**
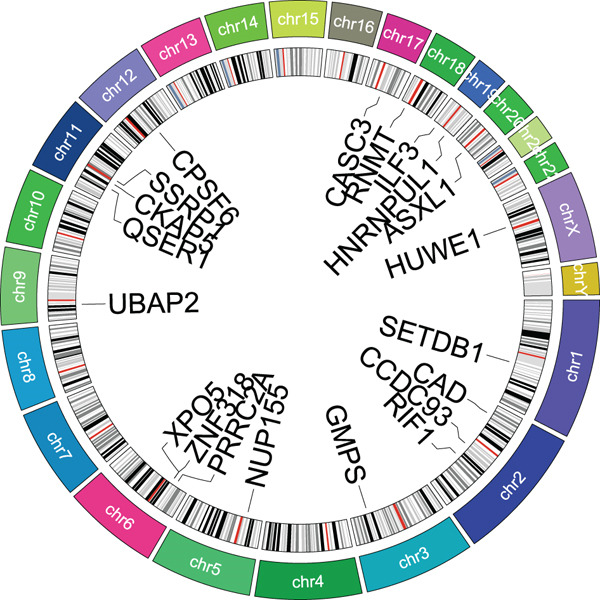
(g)

### 3.2. Selection of Potential Genes and Development of Machine Learning Models

To further investigate prognostically relevant CIN genes in HCC, we performed an intersection analysis of genes obtained from the TCGA and GSE54236 datasets, identifying 73 candidate genes (Figure 1e, Table S5). To better classify and elucidate the roles of these genes, we applied 101 different machine learning algorithms to analyze their expression profiles (Table S1). Most models achieved a (C‐index) of 0.7 or higher, indicating that the selected gene set is significantly associated with HCC prognosis. Based on the average c‐index across TCGA and GSE54236 datasets, we selected the StepCox[both] combined with CoxBoost model for further analysis (Figure 1f). Using this approach, 20 CIN‐related genes were identified as key prognostic markers (Figure 1g, Table S8).

### 3.3. Validation of the Risk Model

We combined results from StepCox and CoxBoost algorithms to create a composite risk score. Patients were divided into high‐ and low‐risk groups using the median score as the cutoff. Survival analysis revealed that the high‐risk group had significantly poorer overall survival (Figure 2a,e). The model′s predictive ability was assessed by ROC curves, showing 5‐year survival AUC values above 0.7 in both TCGA and GSE54236 cohorts (Figure 2b,f), indicating good accuracy. This integrated model outperformed traditional clinical staging in prediction (Figure 2c,d). Both univariate and multivariate Cox regression analyses confirmed that the risk score serves as an independent predictor of prognosis, maintaining high HRs after adjusting for age, gender, and stage (Figures 2i, 2j, 2k, and 2l). Validation with independent cohorts from two centers confirmed the model′s strong accuracy and stability in predicting survival of HCC patients.

Figure 2(a, e) Survival curves of high/low risk groups in TCGA and GSE54236 datasets. (b, f) ROC curves for 1/3/5‐year survival prediction. (c, d) ROC comparison between the model and clinical indicators. (i–l) Univariate and multivariate Cox forest plots.

**Figure:**
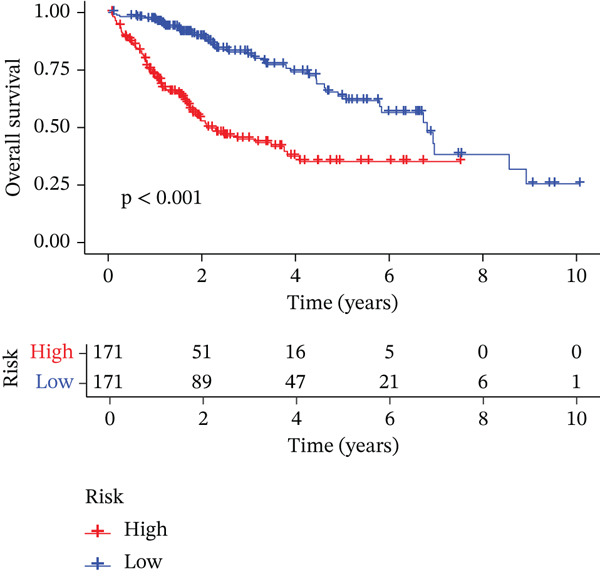
(a)

**Figure:**
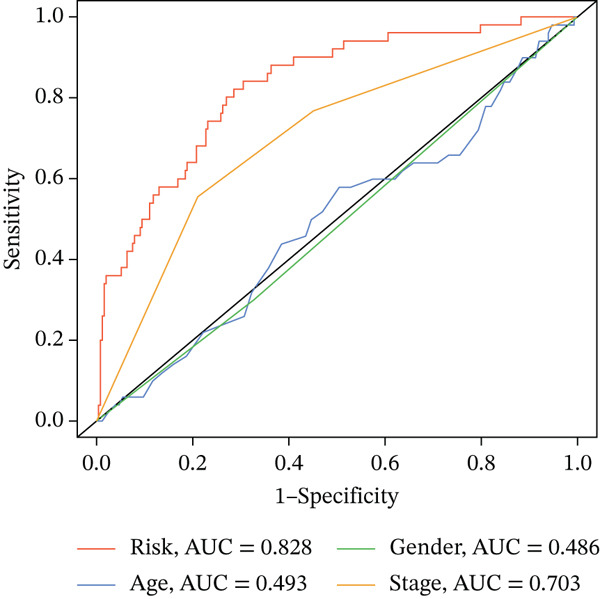
(b)

**Figure:**
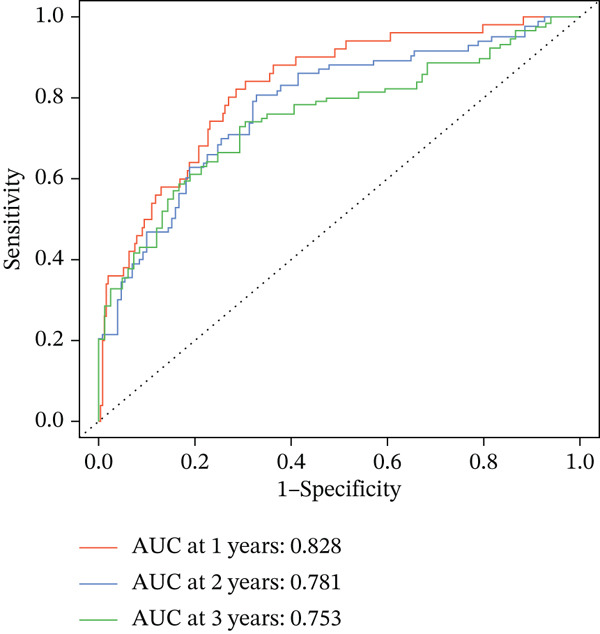
(c)

**Figure:**
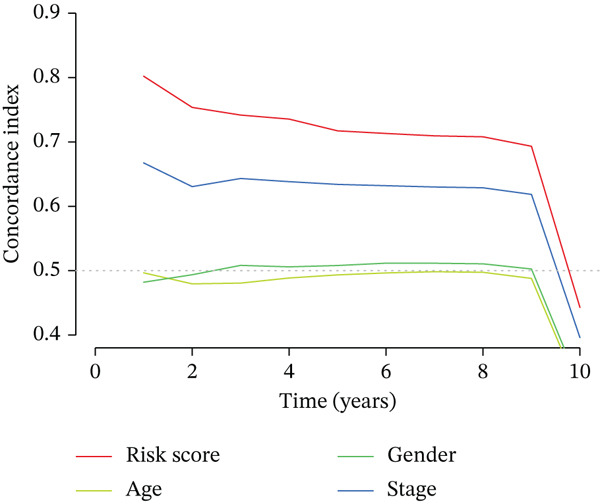
(d)

**Figure:**
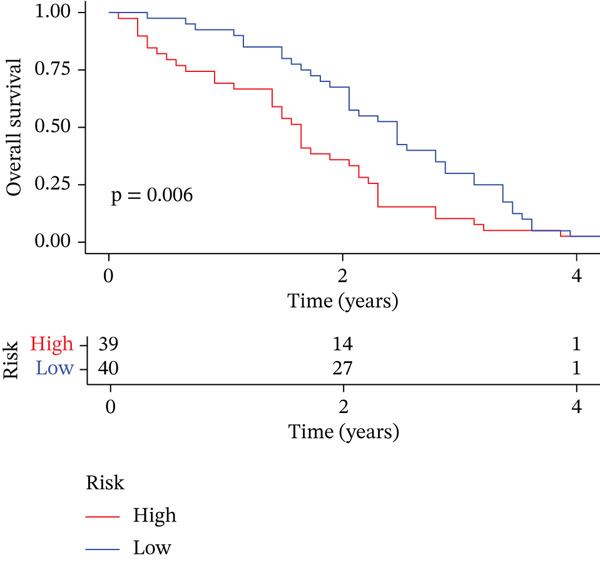
(e)

**Figure:**
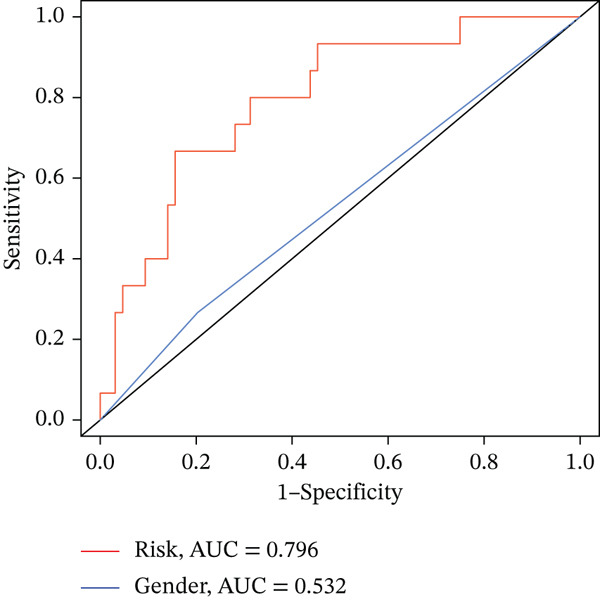
(f)

**Figure:**
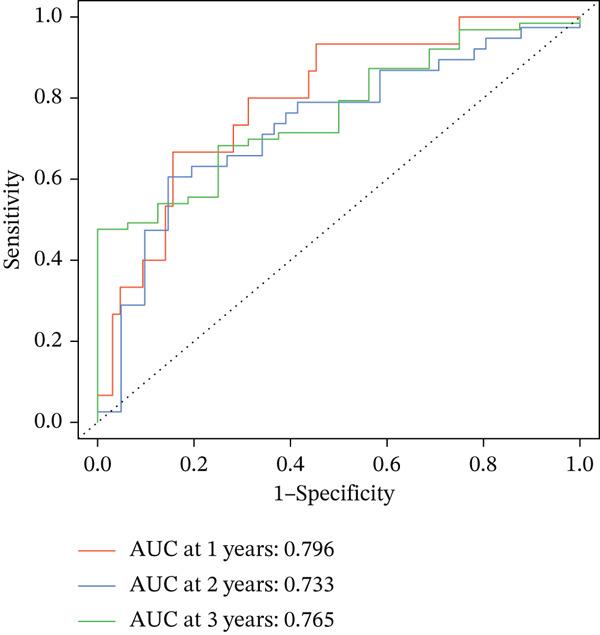
(g)

**Figure:**
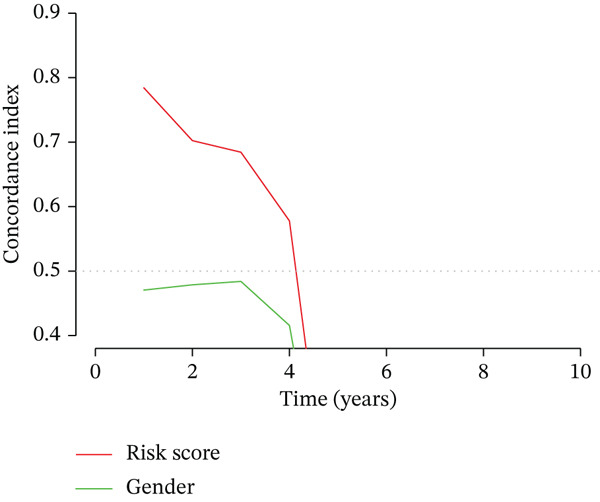
(h)

**Figure:**
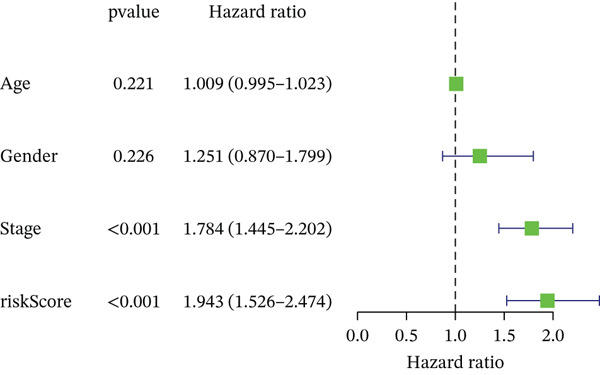
(i)

**Figure:**
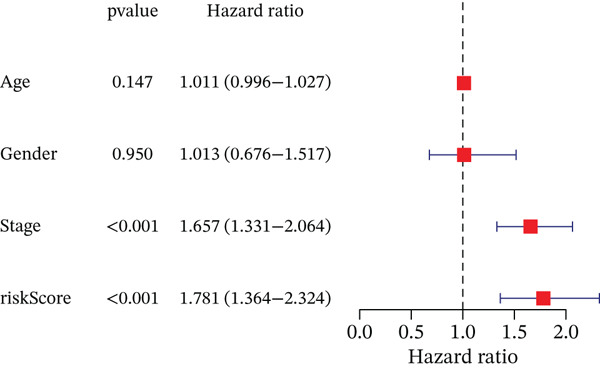
(j)

**Figure:**
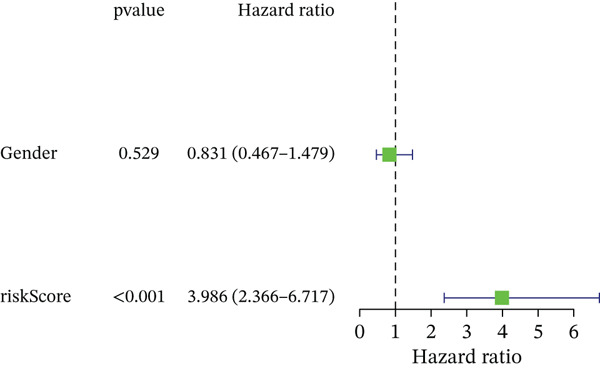
(k)

**Figure:**
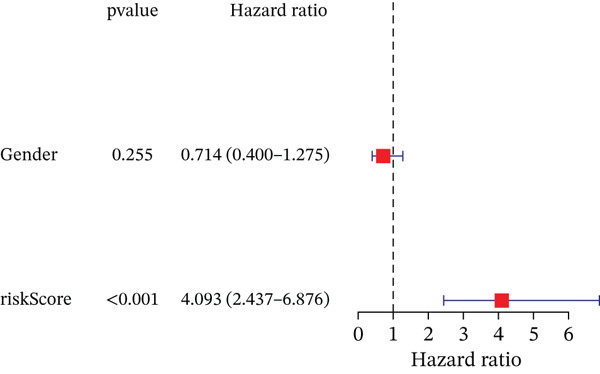
(l)

### 3.4. Differential Signaling Pathway Analysis

Using TCGA transcriptomic data, we identified gene sets differentially expressed between high‐ and low‐risk groups and performed pathway annotation. GO analysis showed that genes upregulated in the high‐risk group were enriched in processes related to tumor progression, such as “cell cycle regulation” and “DNA damage repair.” In contrast, the low‐risk group was enriched in pathways involved in hepatocyte homeostasis, including “amino acid metabolism” and “lipid metabolism” (Figure 3a). KEGG analysis supported these results: high‐risk samples activated pathways like “cell cycle,” “DNA replication,” and “mismatch repair,” whereas low‐risk samples were associated with “primary bile acid biosynthesis” and “amino acid degradation” (Figure 3b). Hallmark enrichment revealed upregulation of the G2/M checkpoint and E2F targets in the high‐risk group, indicating increased proliferation, which was absent in the low‐risk group (Figure 3c). Overall, the two risk subgroups differ in core molecular pathways: the high‐risk group relies on replication stress repair and cell cycle progression, whereas The low‐risk group focuses on metabolic homeostasis, shedding light on HCC heterogeneity.

Figure 3(a) GO enrichment bubble plot. (b) KEGG enrichment heatmap. (c) Hallmark enrichment results.

**Figure:**
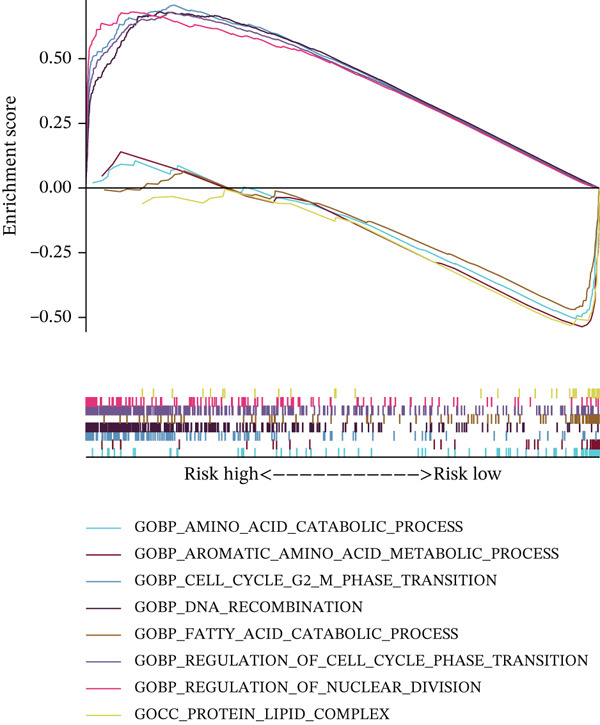
(a)

**Figure:**
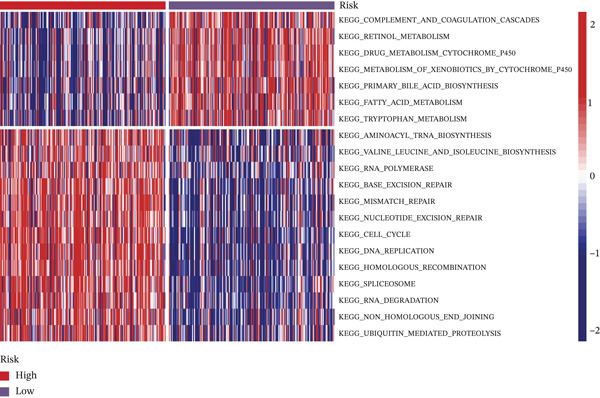
(b)

**Figure:**
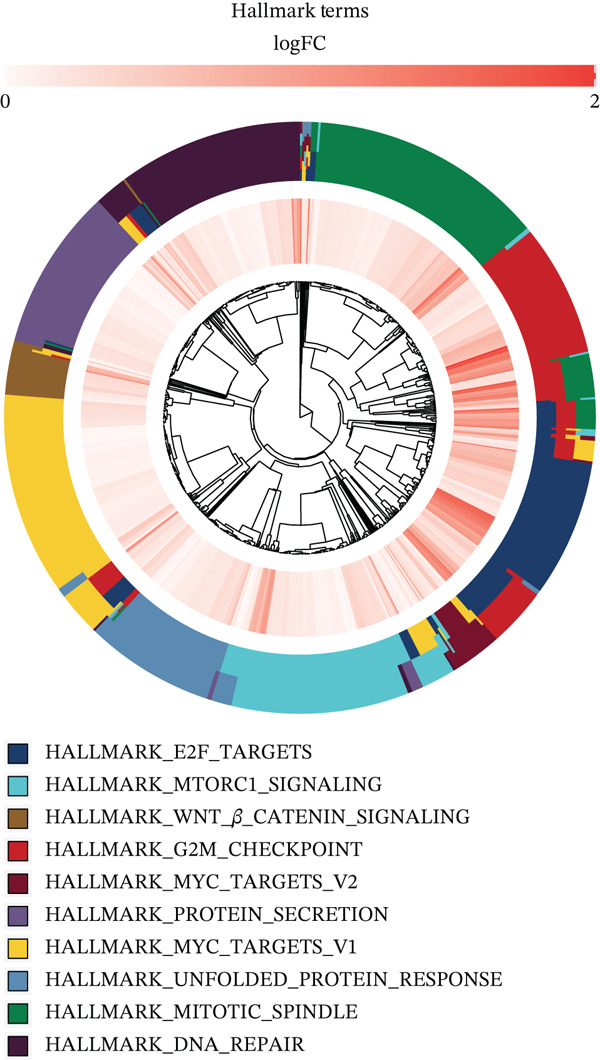
(c)

### 3.5. Differential Immune‐Related Signaling Pathways

Utilizing ssGSEA, we took a deep dive into immune‐related signaling pathways across high‐risk and low‐risk cohorts, uncovering substantial disparities in immune signaling expression. With respect to signaling pathways implicated in immune cell infiltration and the advancement of tumorigenesis (Figure 4a), genes responsible for antigen presentation, costimulation, and cytokine activity showed strikingly different expression profiles between these groups. These findings hint at the possibility that immune regulatory mechanisms within the tumor microenvironment of high‐risk HCC patients might be tilting the scales in favor of tumor initiation and progression. As illustrated in Figure 4b, signaling pathways associated with the cell cycle, DNA replication, and mismatch repair exhibited markedly elevated activity in the high‐risk cohort, suggesting augmented DNA recombination and repair mechanisms that may facilitate tumor invasion, metastatic dissemination, and genomic instability in HCC. Figure 4c illustrates that the low‐risk group demonstrated higher gene expression in macrophage activation and inflammation‐related immune signaling pathways, implying a stronger immune cell activation and inflammatory response that may shape a tumor microenvironment conducive to tumor growth. Ultimately, examination of the seven‐phase cancer immunity cycle (Figure 4d) demonstrated that the high‐risk cohort was predominantly linked to the processes of “cancer cell antigen release” and “immune cell infiltration within tumor microenvironments,” suggesting heightened tumor immune infiltration in patients with high‐risk HCC. Collectively, these findings suggest that immune infiltration correlated with tumor progression is markedly pronounced in the high‐risk group, whereas the low‐risk cohort mainly manifests immune responses indicative of initial stages of tumorigenesis.

Figure 4(a, b, c) ssGSEA heat maps. (d) Comparison of 7‐step cancer immune response scores.

**Figure:**
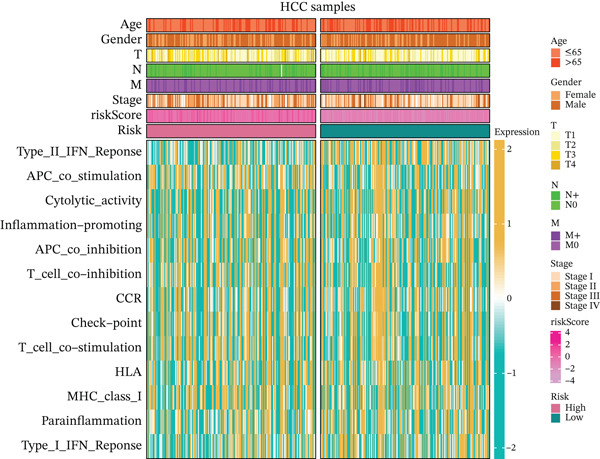
(a)

**Figure:**
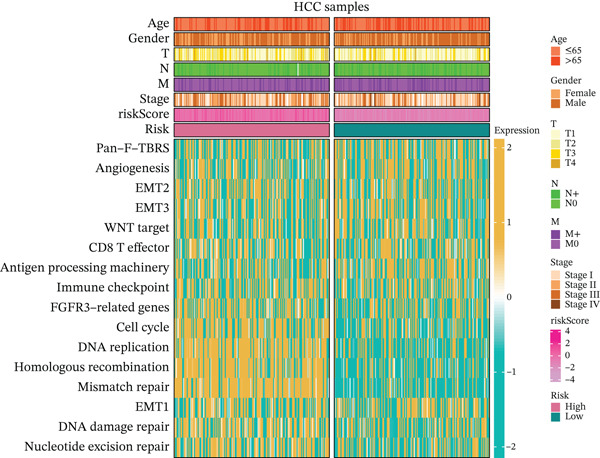
(b)

**Figure:**
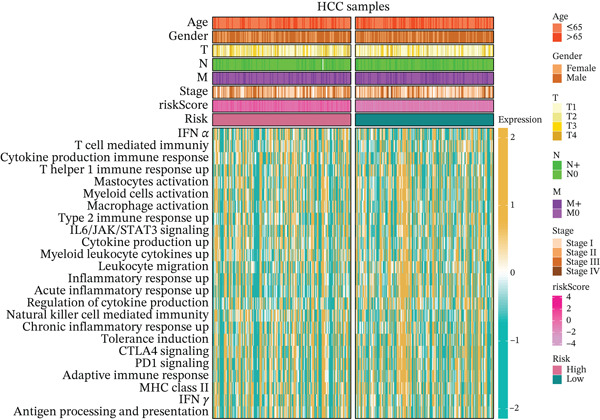
(c)

**Figure:**
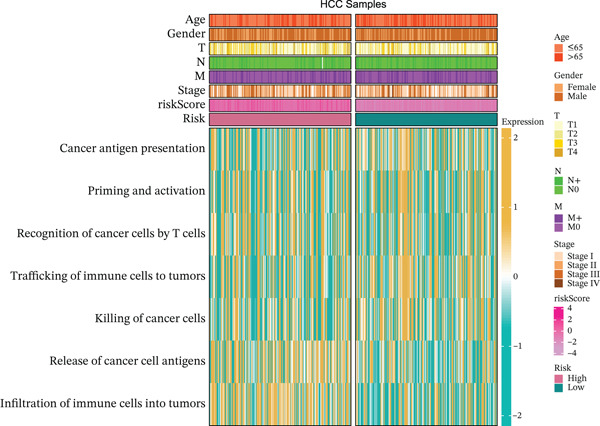
(d)

### 3.6. Composition of Immune Cells and Features of the Tumor Microenvironment

To get a complete picture of the immune cell makeup in both high‐risk and low‐risk patient groups, we put HCC samples through the wringer using six distinct methods for estimating immune infiltration: CIBERSORT, TIMER, xCell, Quantiseq, MCP‐counter, and EPIC. When all was said and done, the data painted a clear picture showing that as risk scores went up, so did the overall presence of T cells in the samples. However, quantification of M2 macrophages varied across methods—xCell suggested a negative correlation, whereas Quantiseq showed a positive trend (Figure 5a), highlighting the limitations of relying on a single algorithm to capture M2 macrophage dynamics. Subsequent ssGSEA analysis revealed that the proportion of activated CD4^+^ T cells positively correlated with risk score, whereas natural killer (NK) cells and eosinophils were negatively correlated (Figure 5b). TIDE platform analysis demonstrated that high‐risk patients exhibited concurrent increases in immune exclusion signals and myeloid‐derived suppressor cell (MDSC) infiltration, indicating a more severe immunosuppressive status and potential resistance to ICIs (Figure 5c). Analysis of immune checkpoint molecule expression showed significant upregulation of inhibitory molecules such as PDCD1, CD47, and CD276 in the high‐risk group, whereas BTN2A1 was highly expressed in the low‐risk group (Figure 5d). From the stromal perspective, high‐risk samples exhibited significantly decreased StromalScore and ESTIMATE scores, negatively correlating with risk score (Figures 5f, 5g, 5h, and 5i), suggesting reduced stromal components and higher tumor purity in the microenvironment. Collectively, the risk score not only reflects variations in T cell abundance but also captures concurrent features of MDSC expansion, checkpoint upregulation, and stromal depletion, providing potential directions for remodeling the immune microenvironment and therapeutic intervention in HCC.

Figure 5(a) Correlation between immune cells and risk score across different algorithms. (b) Clustered heatmap of immune cell types. (c) Chord diagram of immune cells and risk score. (d) Radar plot of immune checkpoint molecule expression. (e) Radar plot of immune regulatory molecule expression. (f) Violin plot of StromalScore in high‐ and low‐risk groups. (g) Scatter plot of the correlation between StromalScore and risk score. (h) Violin plot of ESTIMATEScore in high‐ and low‐risk groups. (i) Scatter plot of the correlation between ESTIMATEScore and risk score.

**Figure:**
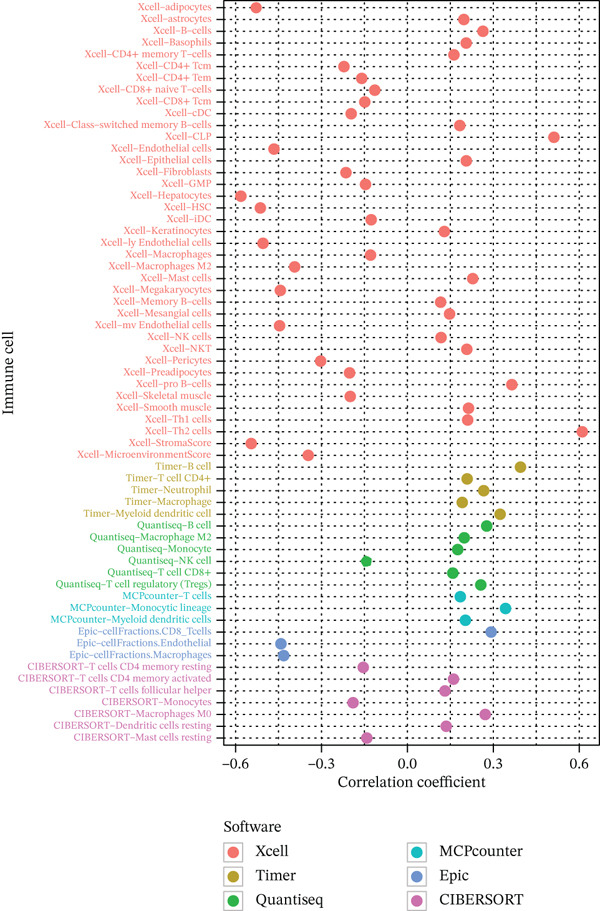
(a)

**Figure:**
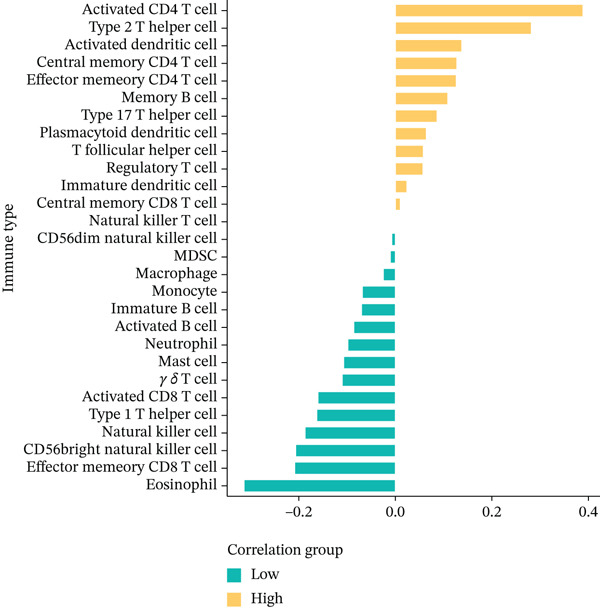
(b)

**Figure:**
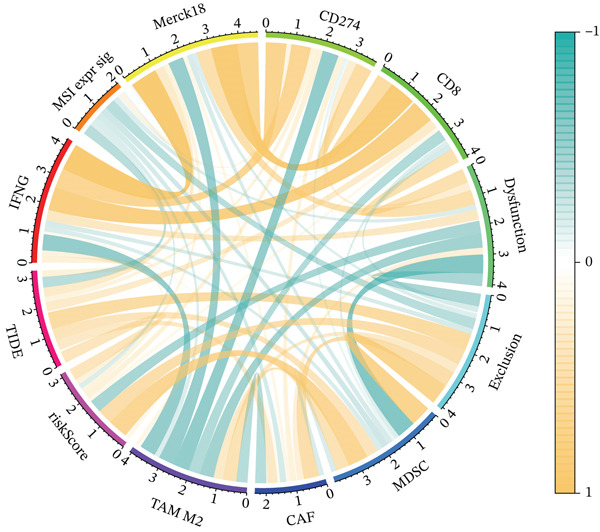
(c)

**Figure:**
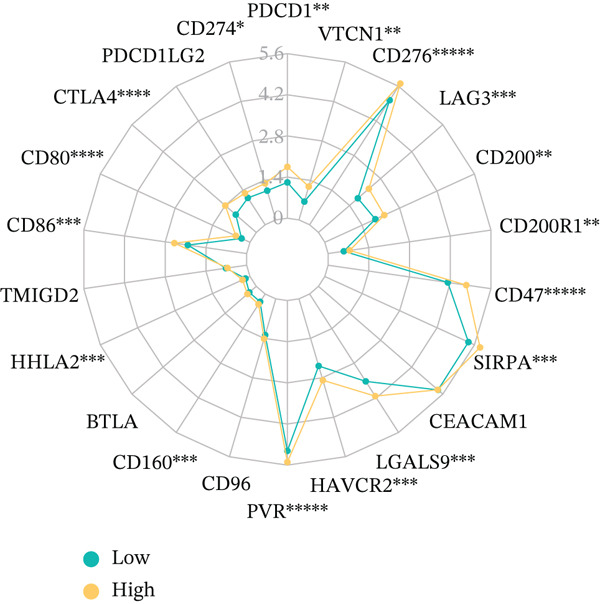
(d)

**Figure:**
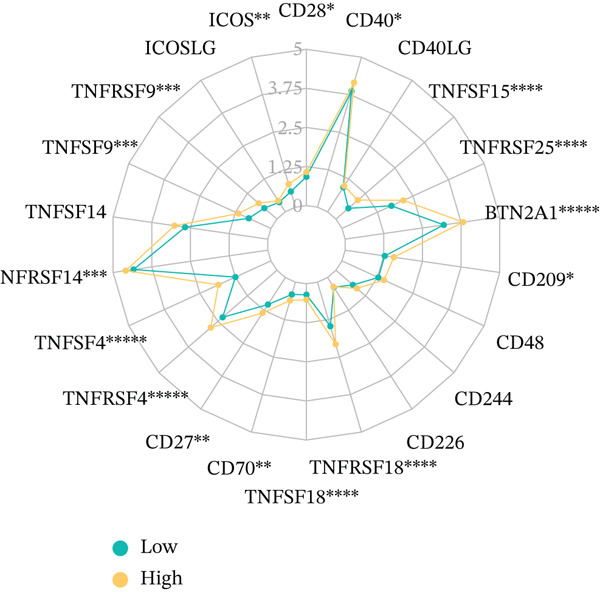
(e)

**Figure:**
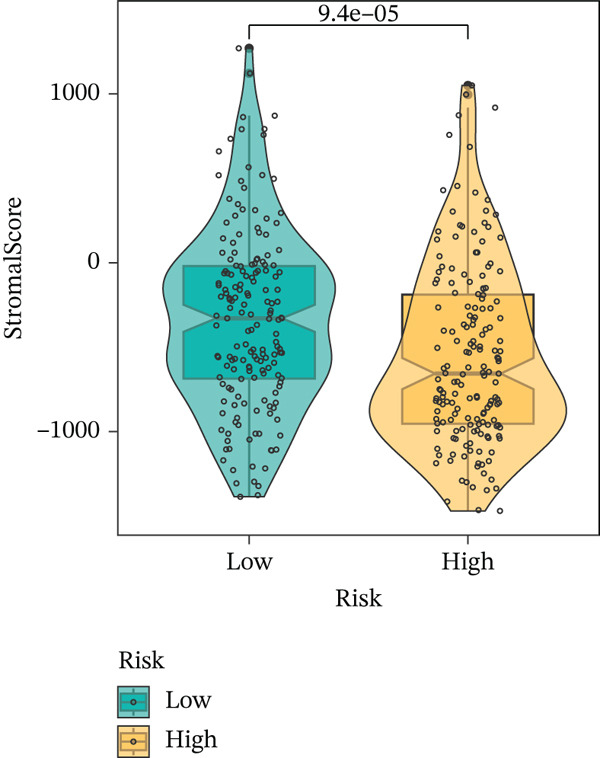
(f)

**Figure:**
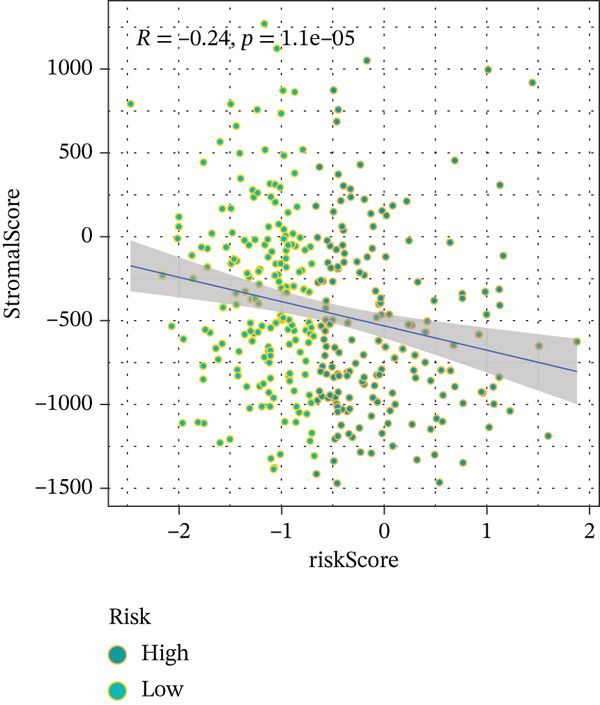
(g)

**Figure:**
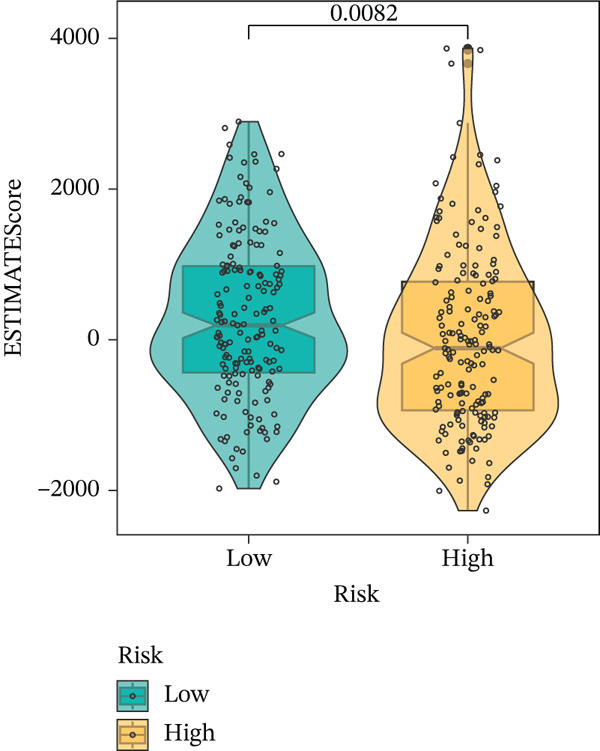
(h)

**Figure:**
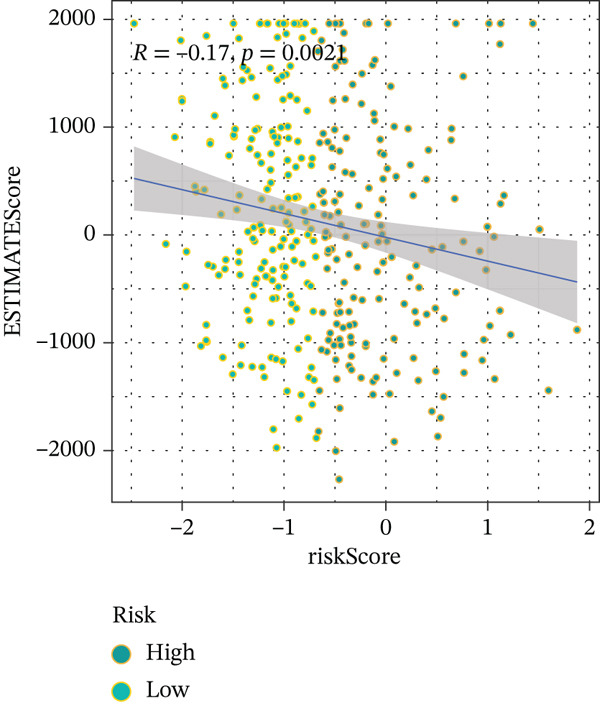
(i)

### 3.7. Clinical and Pathological Characteristics

Comparison of clinical features between high‐ and low‐risk groups (Figure 6a) showed a significantly higher proportion of immunotherapy nonresponders in the high‐risk group, whereas responders were more frequent in the low‐risk group, with nonresponders mainly in Stages II and III. Stratified analysis (Figure 6b) revealed that the low‐risk subgroup had the highest proportion of stage T1 patients, whereas T2 and T3 stages were more prevalent in the high‐risk group. Risk scores showed a positive correlation with tumor stage (Figures 6c, 6d, and 6e), but it should be noted that the median risk score for Stage IV patients exhibited an unexpected decline (Figure 6e). This phenomenon is entirely attributable to the extremely limited number of Stage IV tumors in the TCGA cohort, where the sample size was too small to yield a stable estimate and thus prone to random error. Nevertheless, risk scores in T2, T3, and T4 stages were significantly higher than in T1, indicating a positive correlation between risk scores and tumor stage. Mortality was significantly increased in the high‐risk group, confirming the risk score′s predictive value for patient outcomes and clinical stratification (Figure 6f,g). Tumor mutation burden (TMB) showed no significant difference between groups; however, maximum variant allele frequency (MaxVAF) effectively distinguished the groups and correlated positively with risk scores (Figures 6h, 6i, 6j, and 6k). Collectively, the risk score reflects pathological features and prognosis, suggesting its utility for personalized clinical decision‐making.

Figure 6(a) Comparison of immunotherapy response. (b) T stage distribution. (c–e) Risk scores of different T stages. (f–g) Survival outcomes. (h–k) TMB and MaxVAF.

**Figure:**
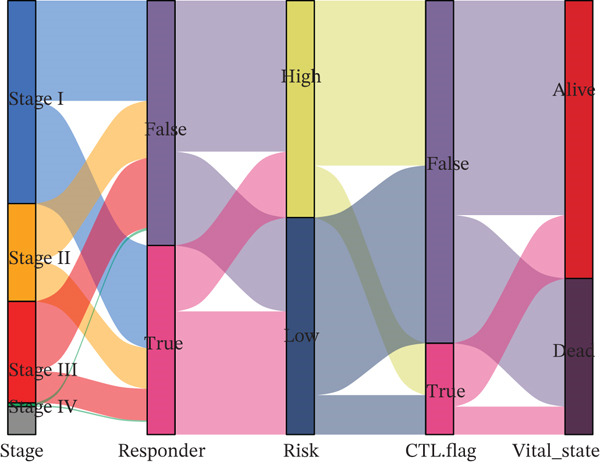
(a)

**Figure:**
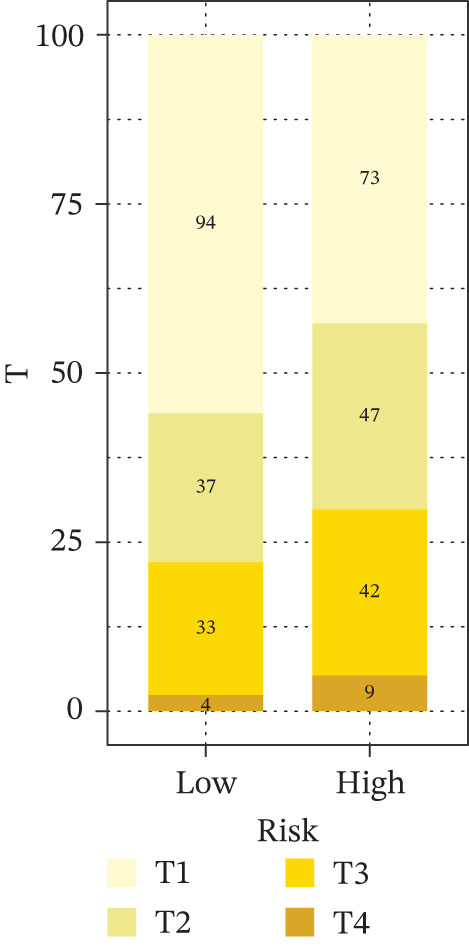
(b)

**Figure:**
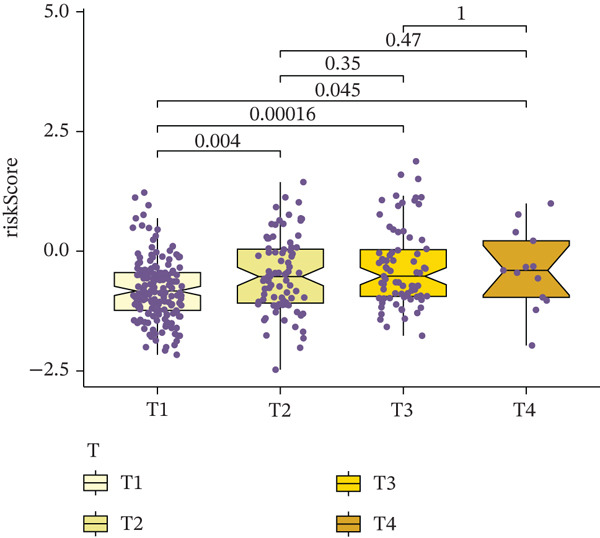
(c)

**Figure:**
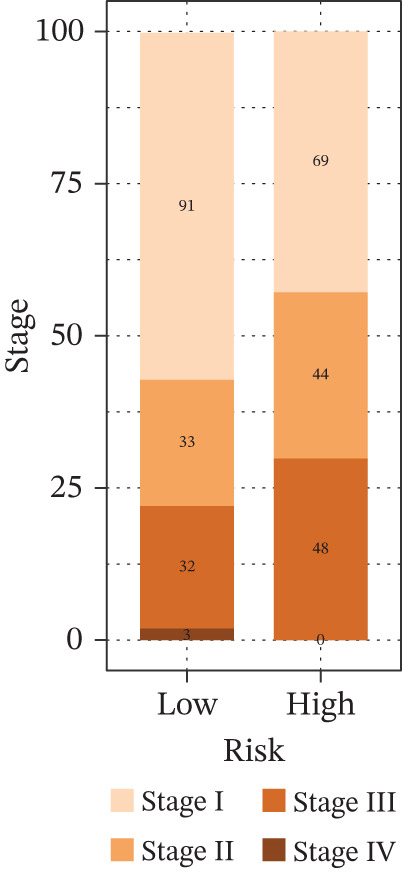
(d)

**Figure:**
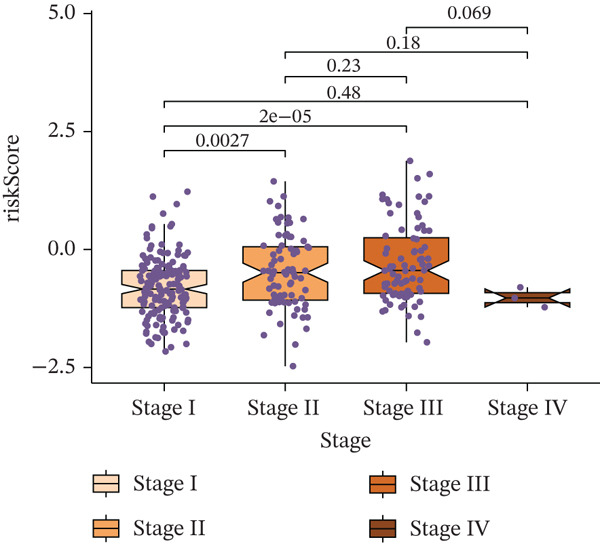
(e)

**Figure:**
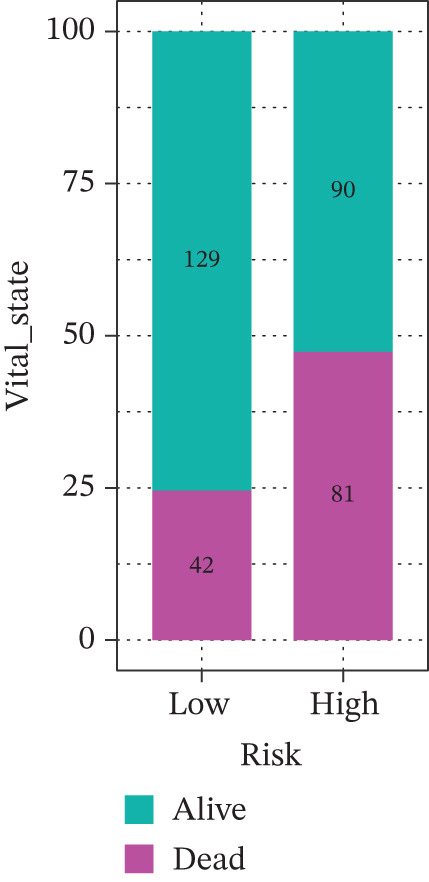
(f)

**Figure:**
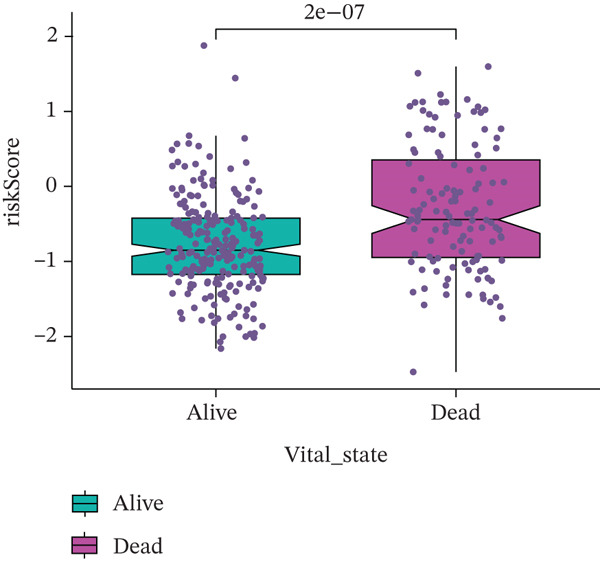
(g)

**Figure:**
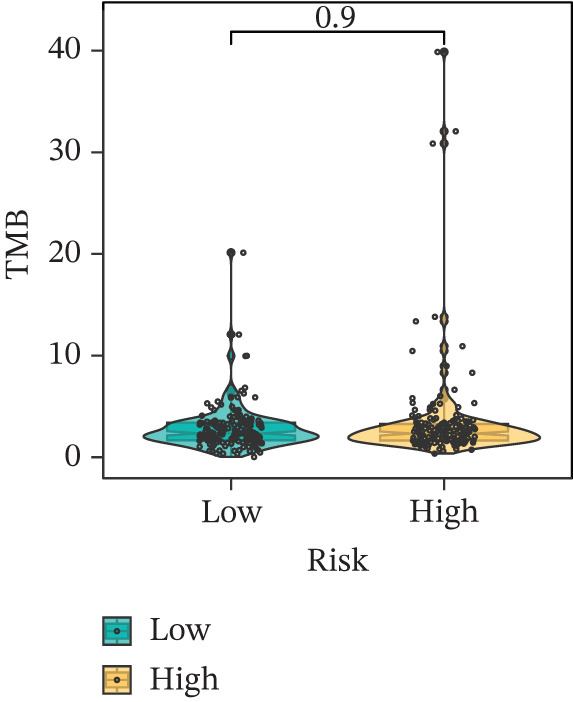
(h)

**Figure:**
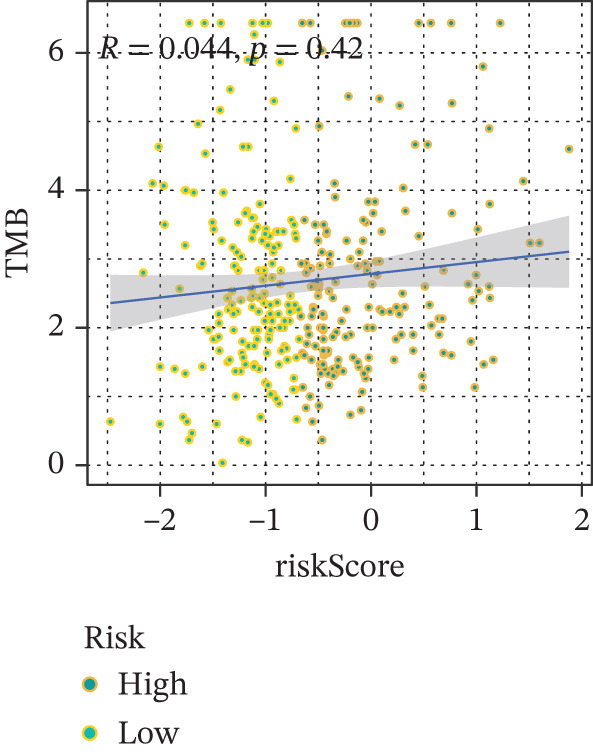
(i)

**Figure:**
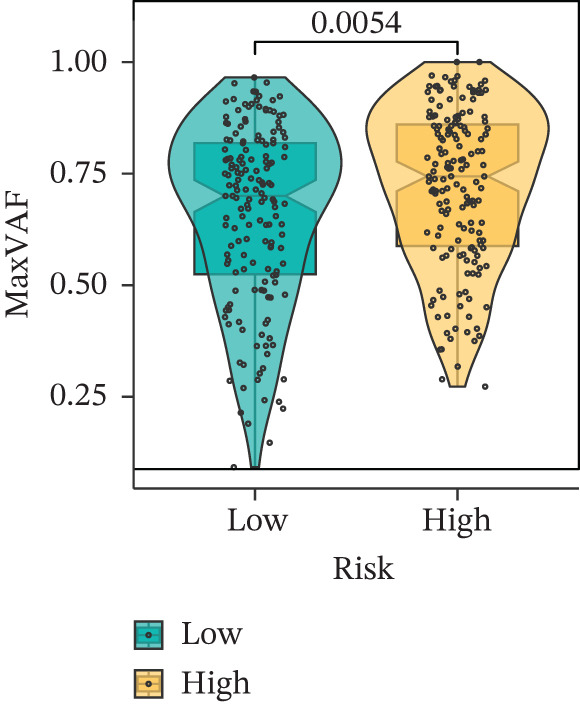
(j)

**Figure:**
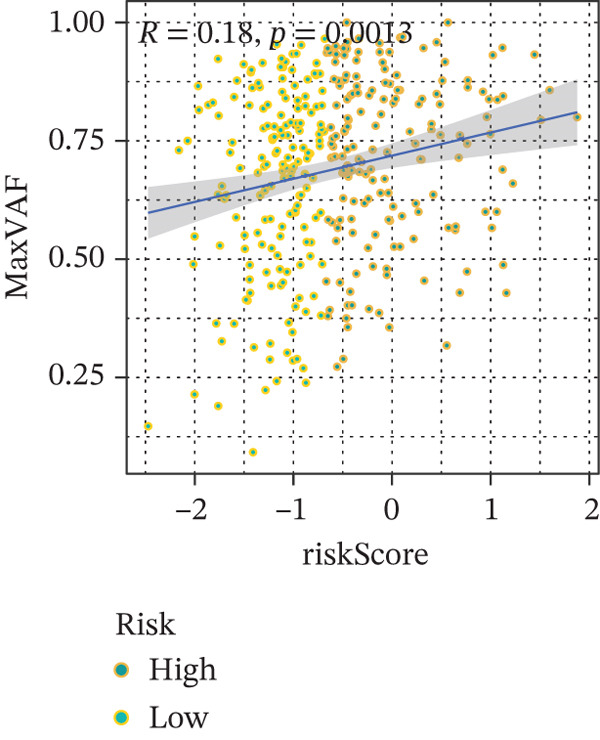
(k)

### 3.8. Genetic Mutation Characteristics

To investigate genetic mutation differences between risk groups, we analyzed mutation frequencies and counts in high‐ and low‐risk cohorts. In TCGA samples, TP53 mutations were predominant in the high‐risk group, with significantly lower TP53 mutation frequency in the low‐risk group (Figure 7a,b). Conversely, CTNNB1 mutations were more frequent in the low‐risk group. Comutation analysis (Figure 7c,d) showed TP53 mutations in the high‐risk group often co‐occurred with OBSCN and PCLO mutations, whereas CTNNB1 in the low‐risk group mainly comutated with ARID2. Variant allele frequency (VAF) analysis (Figure 7e,f) revealed high VAF mutations in the high‐risk group mainly involved RB1 and AXIN1, whereas TP53 and BAP1 mutations dominated in the low‐risk group, indicating clonal mutation differences. Overall, the risk score distinguishes molecular mutation profiles between risk groups, offering valuable insights for precision therapy in HCC.

Figure 7(a, b) Mutation waterfall plots. (c, d) Comutation heatmaps. (e, f) VAF analysis.

**Figure:**
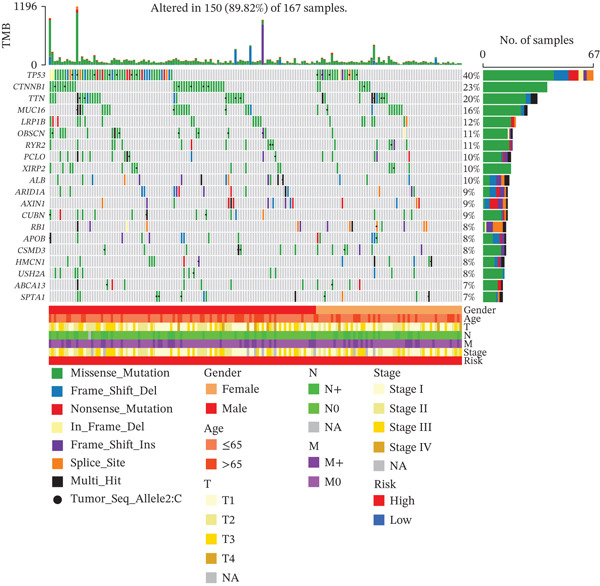
(a)

**Figure:**
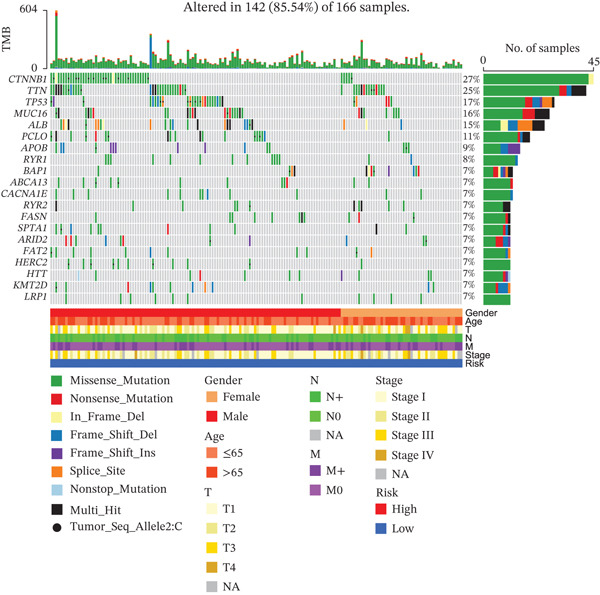
(b)

**Figure:**
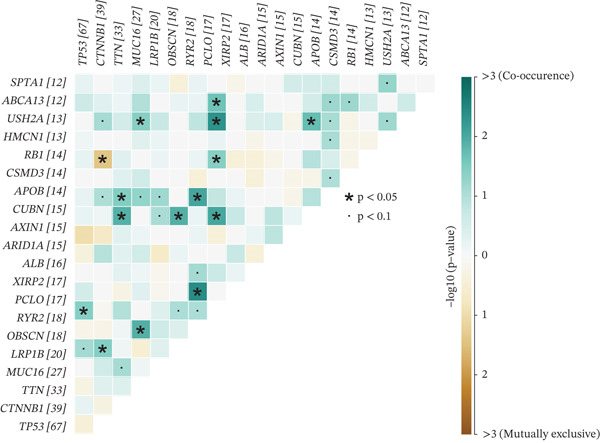
(c)

**Figure:**
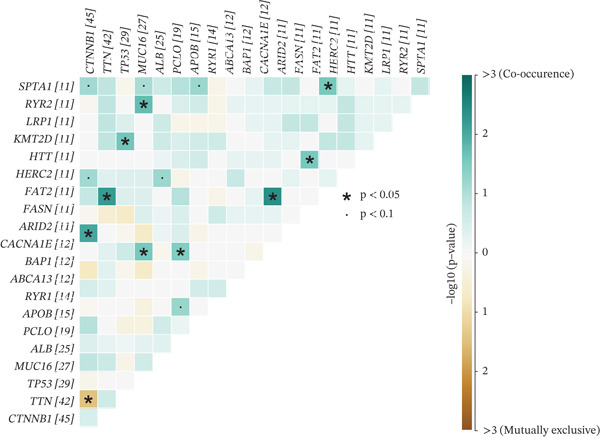
(d)

**Figure:**
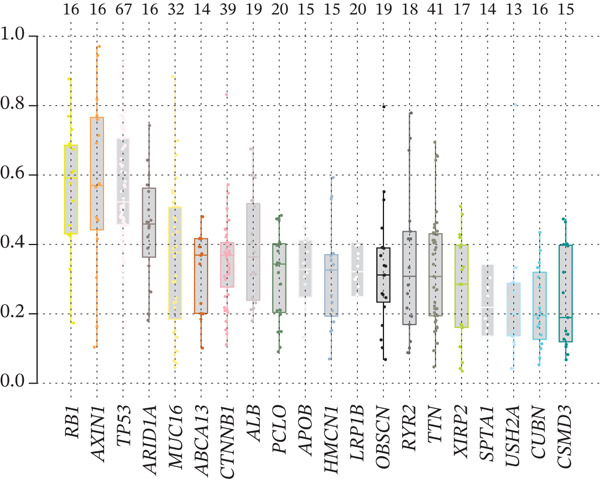
(e)

**Figure:**
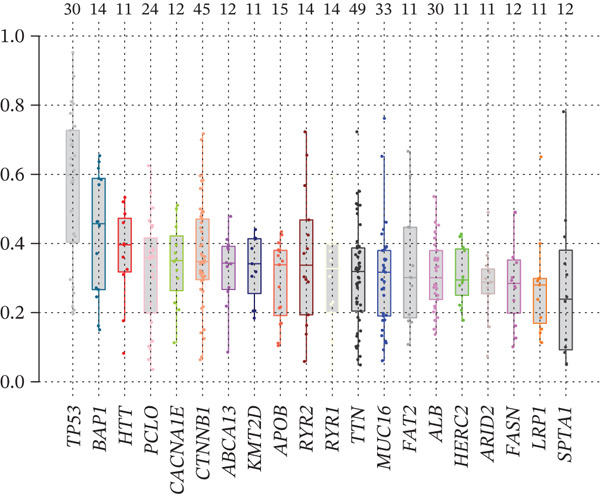
(f)

### 3.9. Drug Sensitivity of the Risk Score

Whether patients can benefit from subsequent chemotherapy depends on their individual sensitivity to chemotherapeutic drugs. In this study, two algorithms, oncoPredict and pRRophetic, were used to calculate the half‐maximal inhibitory concentration (IC50) of six clinically common chemotherapeutic drugs in high‐risk and low‐risk subgroups of HCC. As shown in Figures 8a, 8b, 8c, 8d, 8e, and 8f, the IC50 values of 5‐fluorouracil, paclitaxel, vinorelbine, and dasatinib were significantly reduced in the high‐risk group, which indicated that high‐risk patients had higher sensitivity to the above four drugs. Further negative correlation analysis confirmed that there was an inverse correlation between drug IC50 and risk score (Figures 8g, 8h, 8i, 8j, 8k, and 8l). In contrast, although gemcitabine and cytarabine showed better drug efficacy in the low‐risk group, their IC50 variation trends did not match the risk score, suggesting that the model had limited predictive accuracy for the sensitivity of these two drugs. In conclusion, this risk‐scoring model can effectively reflect the response differences of most chemotherapeutic drugs among different risk subgroups, providing a basis for the preliminary selection of clinical chemotherapy regimens.

Figure 8(a–f) IC50 comparison of 6 chemotherapeutic drugs. (g–l) Correlation between IC50 and risk score.

**Figure:**
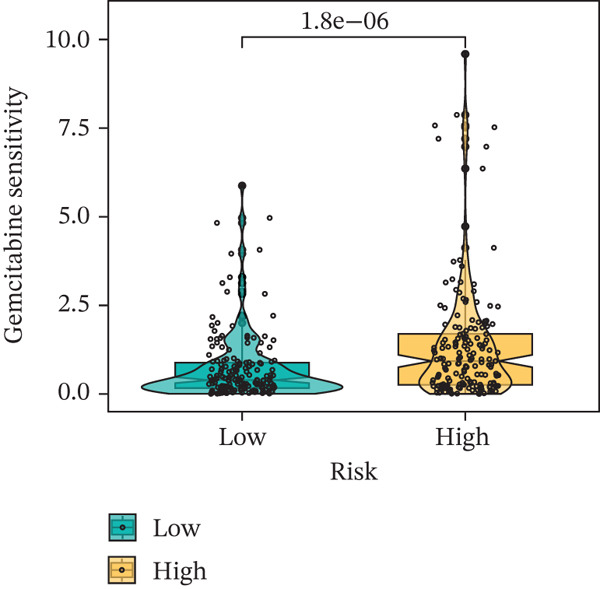
(a)

**Figure:**
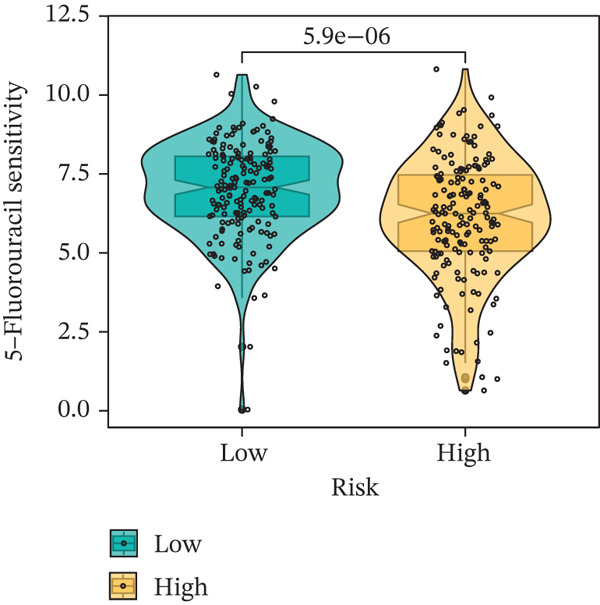
(b)

**Figure:**
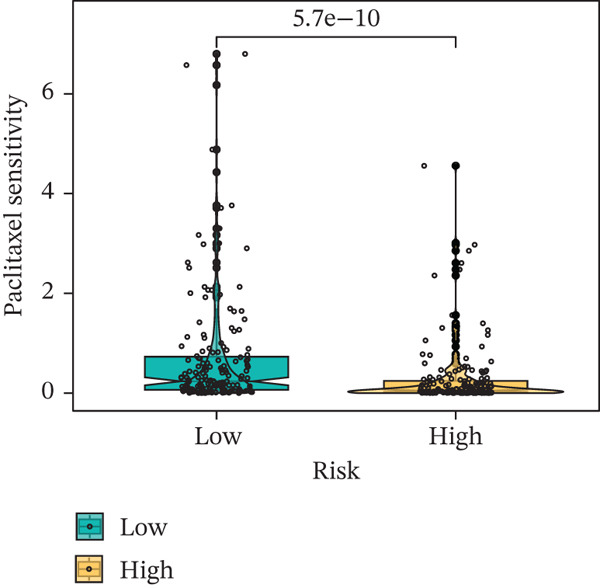
(c)

**Figure:**
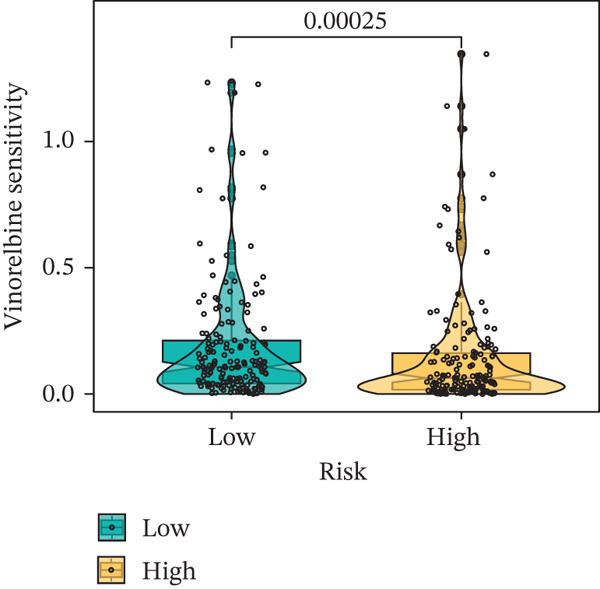
(d)

**Figure:**
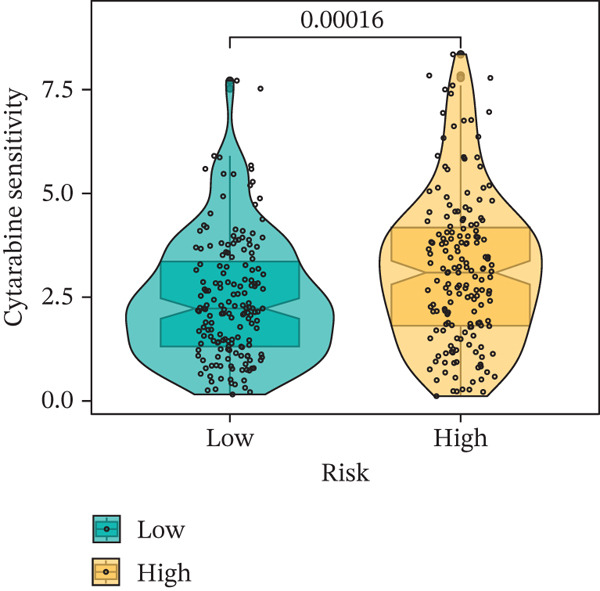
(e)

**Figure:**
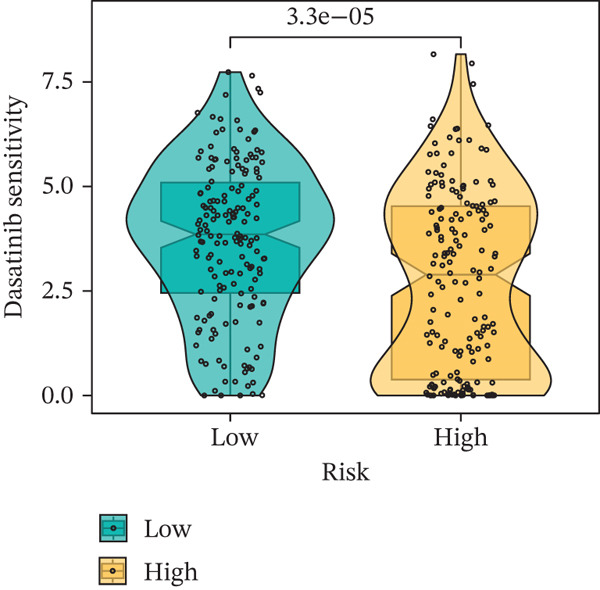
(f)

**Figure:**
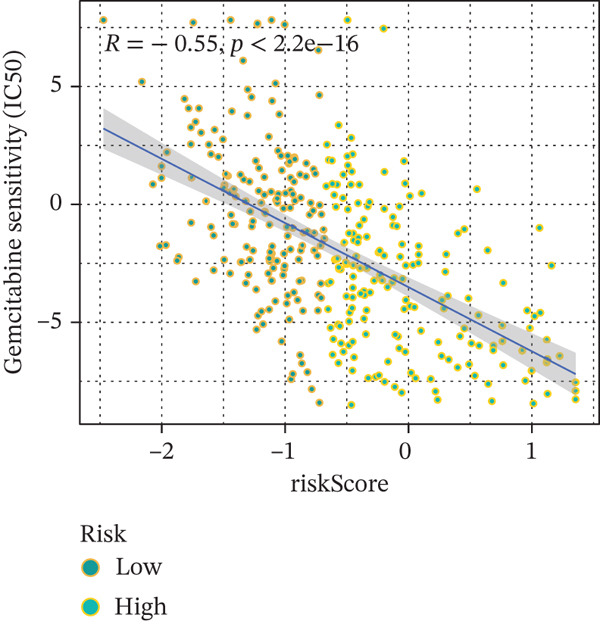
(g)

**Figure:**
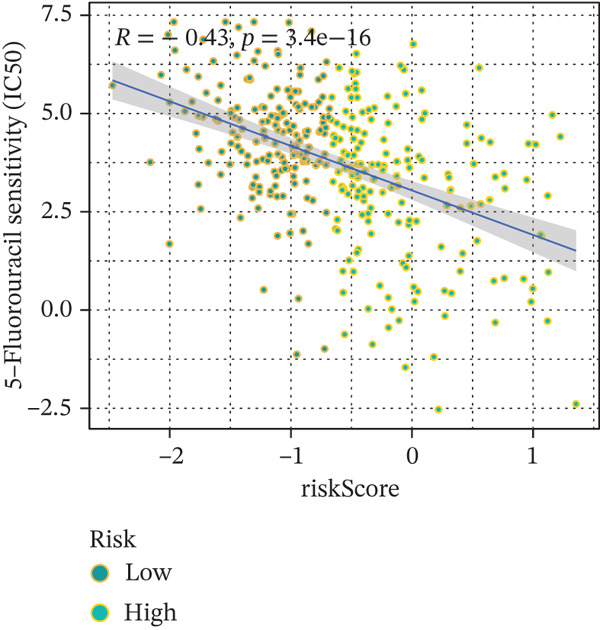
(h)

**Figure:**
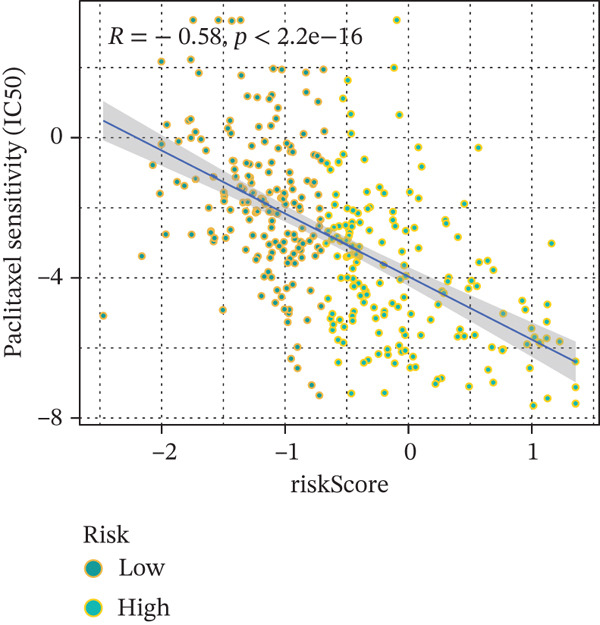
(i)

**Figure:**
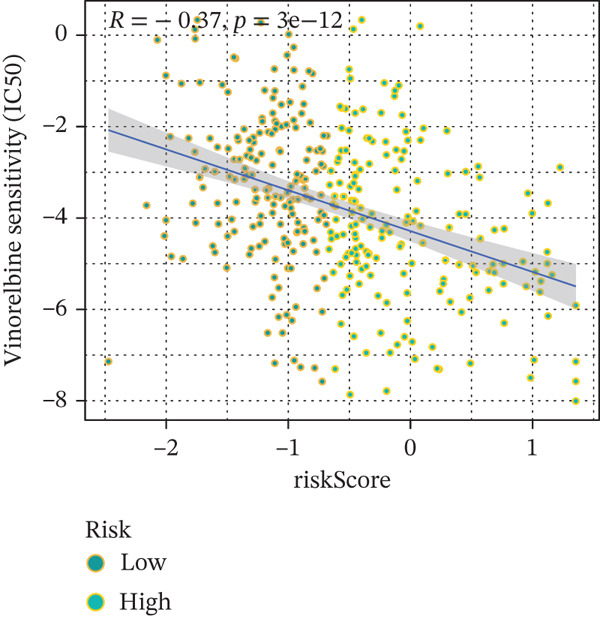
(j)

**Figure:**
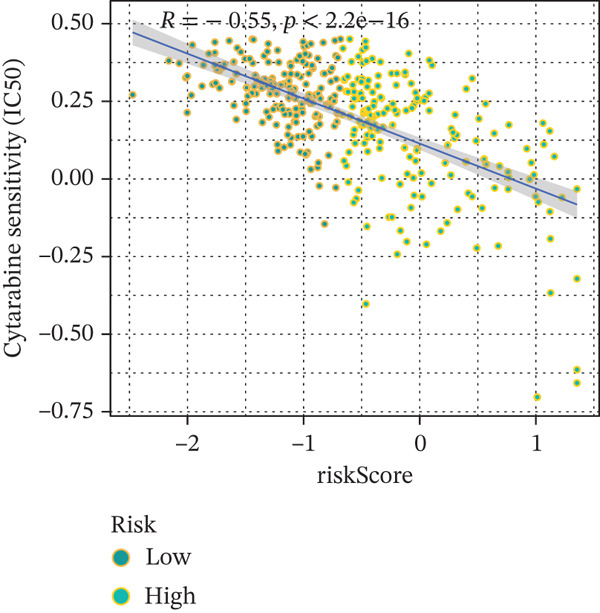
(k)

**Figure:**
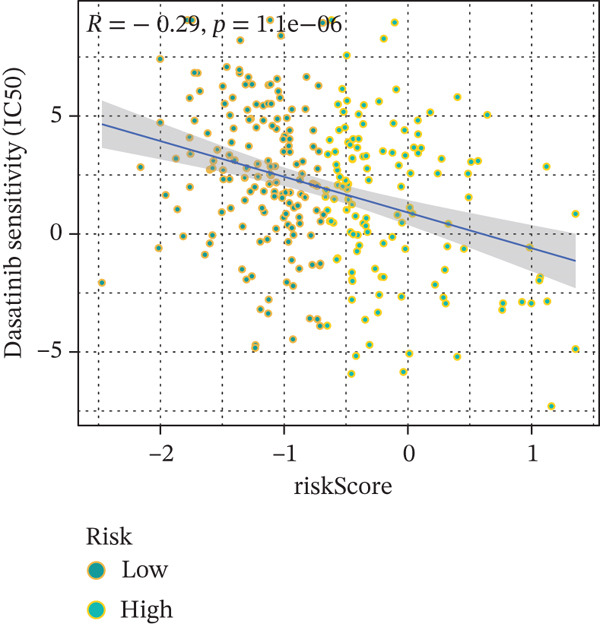
(l)

### 3.10. Prognostic Analysis

We evaluated the prognostic predictive power of the risk model for HCC patients using multivariate Cox regression analysis, adjusting for age, sex, stage, and TMB. The results showed that regardless of whether other clinical features were included, the risk score provided stable and effective reliable prognostic predictions, with a HR of 3.178 (Figure 9a). Further analysis of expression characteristics based on high‐risk and low‐risk groups, as well as TMB stratification, revealed significant differences in prognosis among the groups (Figure 9b). We integrated TMB, clinical staging, and our risk model to develop a comprehensive predictive nomogram (Figure 9c), which accurately forecasted 1‐, 3‐, and 5‐year survival rates. ROC analysis comparing the nomogram, TMB, clinical staging, and risk scores revealed that the nomogram achieved the highest AUC, indicating that combining clinical factors with the risk score substantially improves prediction accuracy.

Figure 9(a) Multivariate Cox forest plot. (b) Combined survival curve stratified by risk score and TMB. (c) Nomogram model. (d) ROC comparison. (e) Calibration curve.

**Figure:**
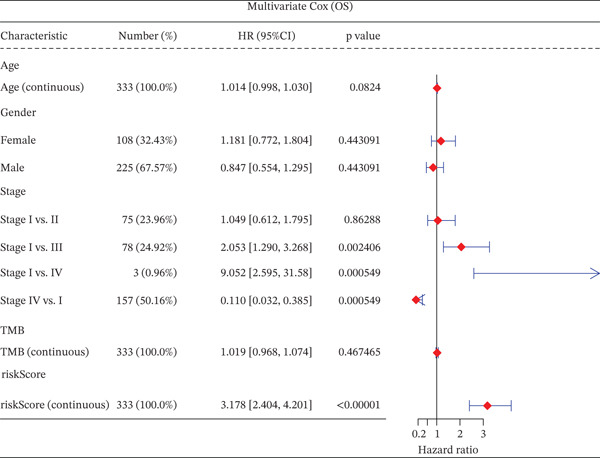
(a)

**Figure:**
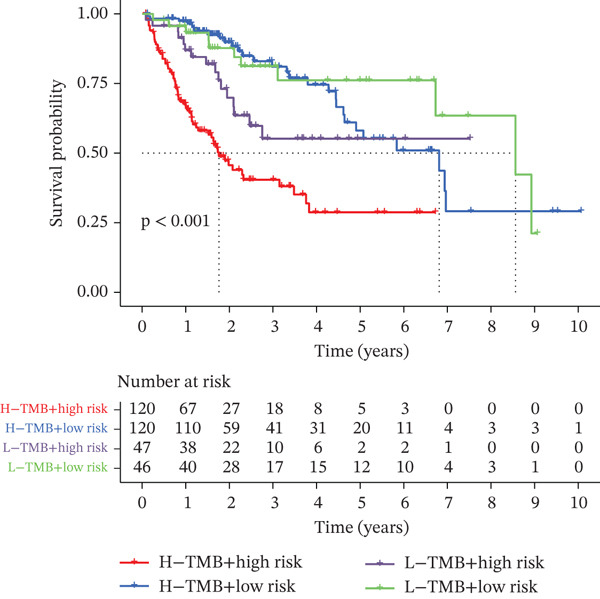
(b)

**Figure:**
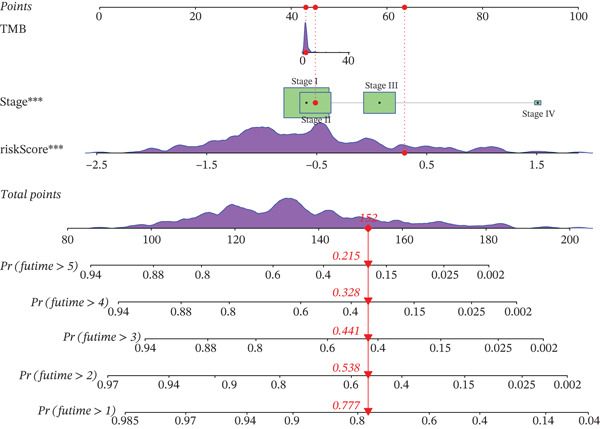
(c)

**Figure:**
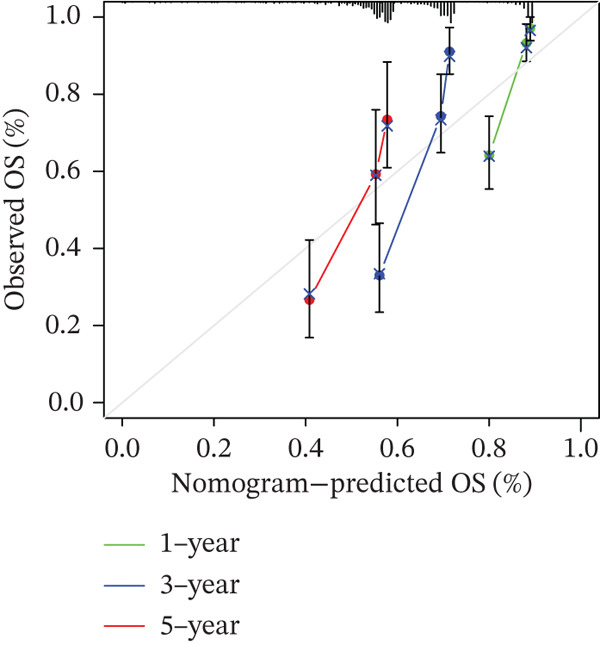
(d)

**Figure:**
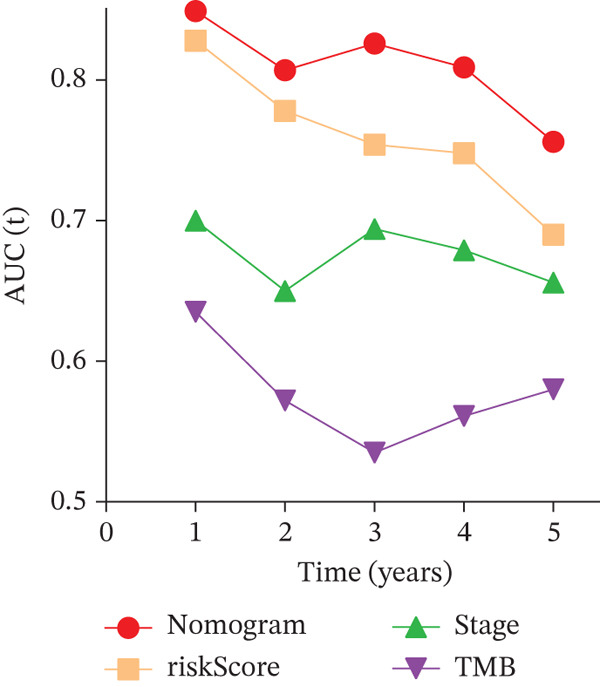
(e)

### 3.11. Functional Validation in Cells

To clarify the biological effects of SSRP1 and SETDB1, we initially screened their expression levels in normal liver cells (MIHA) and four HCC cell lines: H7, 97H, 3B, and PLC. As shown in Figure 10a, both genes were highly expressed in 97H cells, which were thus selected for subsequent functional studies. After siRNA‐mediated knockdown, the target protein levels were significantly reduced, confirming the effectiveness of the intervention (Figure 10b,c). Transwell assays demonstrated that gene knockdown markedly decreased the number of invasive 97H cells crossing the membrane (Figure 10d); EdU incorporation assays further confirmed a significant inhibition of cell proliferation (Figure 10e). In summary, SSRP1 and SETDB1 play important roles in regulating proliferation and invasion of HCC cells and may serve as potential molecular targets for HCC therapy.

Figure 10(a) Protein expression in liver cell lines. (b, c) Validation of siRNA interference efficiency. (d) Transwell invasion assay. (e, f) EdU proliferation assay.

**Figure:**
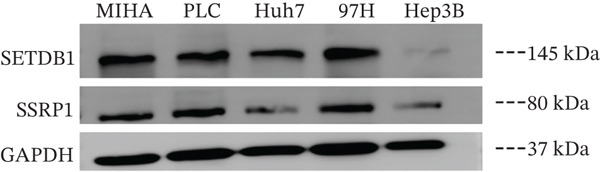
(a)

**Figure:**
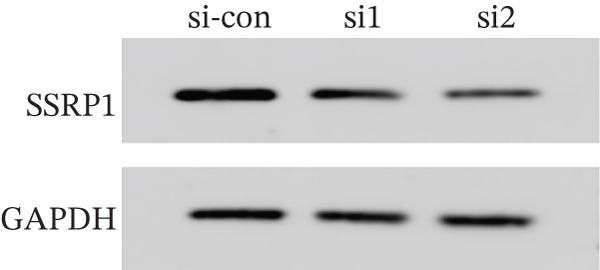
(b)

**Figure:**
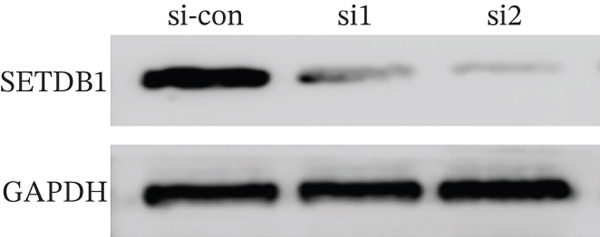
(c)

**Figure:**
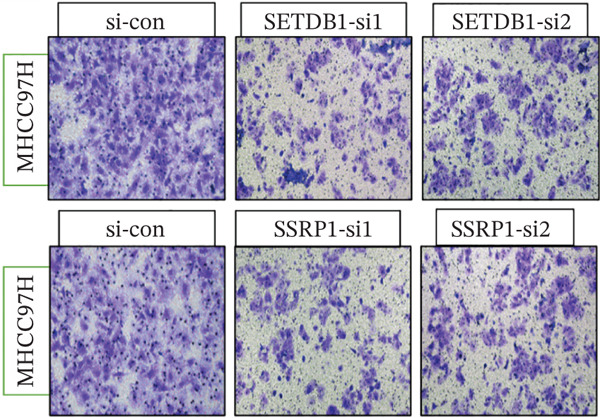
(d)

**Figure:**
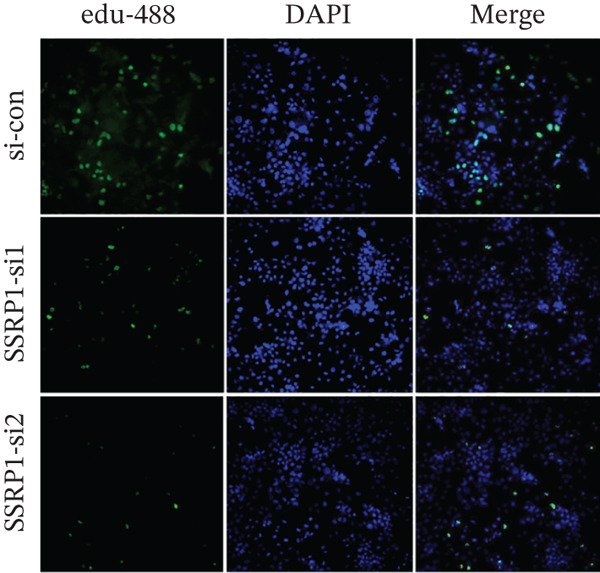
(e)

**Figure:**
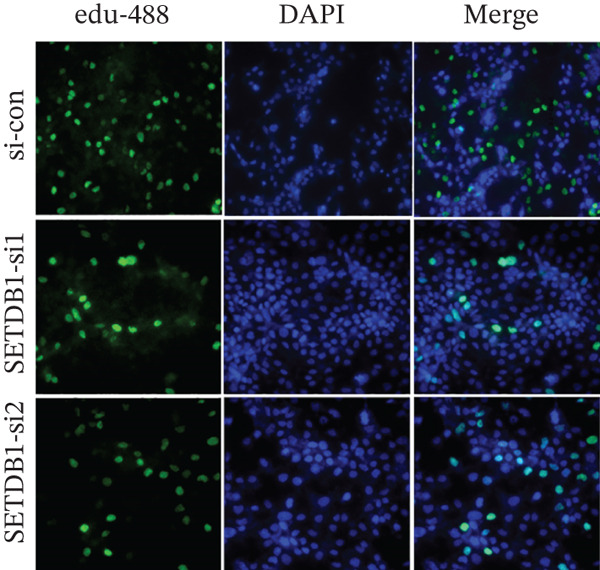
(f)

## 4. Discussion

CIN means abnormal chromosome separation during cell division, causing changes in chromosome number or structure, and is a key feature of tumors [10], In this study, CIN varies across risk groups, offering important clues about tumor progression and clinical classification. In the high‐risk group, TP53 mutations often occur together with OBSCN and PCLO mutations. Loss of TP53 function disrupts cell cycle checkpoints, causing buildup of abnormal hepatocytes and centrosome problems, which lead to chromosome missegregation and CIN. This agrees with previous findings that “p53 mutations worsen genomic instability by faulty checkpoint systems” [11, 12]. The low‐risk group mainly shows CTNNB1‐ARID2 comutations. ARID2, part of a chromatin‐remodeling complex, when inactive, may control genome stability differently, resulting in lower CIN levels. CIN status is linked to HCC malignancy and microenvironment. High CIN in the high‐risk group associates with an immunosuppressive environment (more neutrophils and MDSCs), poor outcomes, and sensitivity to certain chemotherapy drugs, showing CIN drives tumor diversity and immune escape. Apart from changes in the number of immune cells, the micronuclear DNA induced by CIN can be recognized by the cGAS‐STING pathway [13]. In high‐CIN tumors, persistent STING signaling induces the secretion of *β*‐interferon (IFN‐*β*) and the chemokine CXCL10, which initially recruits CD8^+^ T cells [14]. However, subsequent chronic activation of nuclear factor *κ*B (NF‐*κ*B) promotes the upregulation of programmed death‐ligand 1 (PD‐L1) and the expansion of MDSCs [15]. In HCC, TP53 deficiency further exacerbates cytoplasmic DNA leakage, thereby enhancing STING‐mediated immune exhaustion and correlating with resistance to anti‐PD‐1 therapy [16]. Therefore, CIN reshapes the immune microenvironment of HCC via the “double‐edged sword” cGAS‐STING axis, providing a theoretical basis for the combination therapy of STING inhibitors and ICIs. Also, high MaxVAF in this group relates to CIN genes like RB1, suggesting gene imbalance helps tumor clones grow. In summary, CIN differences in HCC risk groups are tied to mutation patterns, with TP53 linked to high CIN and CTNNB1‐ARID2 to low CIN. CIN can be a marker for risk and its related pathways (like DNA repair and cell cycle) may be targets for precise treatment, offering new ways to improve diagnosis and therapy.

The immune microenvironment of HCC is a complex regulatory network composed of various immune cells, cytokines, and molecular signals, and its heterogeneity profoundly influences tumor initiation, progression, and therapeutic responses [17]. From a cellular standpoint, the high‐risk group in this study shows elevated infiltration of classic immunosuppressive cells, including neutrophils and MDSCs. These cells contribute to an immunosuppressive environment by releasing cytokines like IL‐10 and TGF‐*β* [18]. Moreover, the high‐risk group exhibits a significant increase in regulatory T cells (Tregs), which suppress the antitumor function of effector T cells. At the molecular level, this group also shows elevated expression of immune checkpoint molecules like PD‐L1. This upregulation is closely linked to CIN caused by TP53 mutations—genomic instability leads to greater tumor antigen heterogeneity, driving tumor cells to increase immune checkpoint expression to escape immune surveillance. This aligns with previous studies that elucidate the mechanism of “p53 mutations inducing immune evasion by interfering with antigen presentation and immune recognition pathways” [19]. In contrast, the low‐risk group displays a more active antitumor immune environment, characterized by higher infiltration of CD4^+^ and CD8^+^ T cells and lower levels of immune checkpoint molecules. This aligns with a relatively stable genome and an immune‐activated phenotype driven by CTNNB1‐ARID2 comutations.

T cells, as important adaptive immune cells in the tumor microenvironment, have a dual role in HCC progression [20]. On one side, active T cells, especially cytotoxic CD8^+^ T cells, kill tumor cells by recognizing their antigens and releasing molecules like perforin and granzymes. They also produce cytokines such as interferon‐*γ* (IFN‐*γ*) and tumor necrosis factor‐*α* (TNF‐*α*), which stop tumor growth and attract other immune cells [21–23]. On the other side, the HCC environment can cause T cells to become exhausted. These exhausted T cells have high levels of checkpoint proteins like PD‐1 and CTLA‐4, lose their ability to kill tumors, and release inhibitory cytokines like IL‐10 and TGF‐*β*, which help tumors avoid the immune system [24]. This study found more T cells in HCC tissues, linked to worse tumor stage and higher death risk, showing that T cell imbalance may help create an immune‐suppressive environment. T cells also interact with tumor fibroblasts (CAFs) and Tregs, weakening immune defense and speeding up HCC progression. So, restoring T cell function or blocking exhaustion pathways may improve HCC immunotherapy.

Immune checkpoint molecules play a central regulatory role in immune evasion and therapeutic response in HCC [25], and the results of this study provide important evidence for understanding their clinical value. Analysis showed that the high‐risk group had a notably higher rate of nonresponders to immunotherapy, closely linked to the expression patterns of immune checkpoint molecules. The high‐risk group was frequently accompanied by high expression of immune checkpoint genes such as PD‐L1, consistent with previous studies reporting differential immunotherapy benefits in patients with high PD‐L1 expression [26, 27]. Differences in immune checkpoint expression are related to tumor mutation profiles and microenvironment features [28]: CIN driven by frequent TP53 mutations in the high‐risk group may indirectly upregulate immune checkpoint expression by enhancing tumor antigen heterogeneity. At the same time, greater infiltration of immunosuppressive cells like neutrophils enhances the immunosuppressive effects driven by checkpoint pathways. By contrast, CTNNB1‐ARID2 comutations characterize the low‐risk group, which exhibits low immune checkpoint expression and better therapeutic response.

Chemotherapy and targeted therapy are key systemic approaches for HCC, with their varied efficacy mainly due to tumor molecular complexity and the dynamic microenvironment, which supports optimizing personalized strategies [29]. From the chemotherapy perspective, differences in drug sensitivity are not only related to classical DNA damage response pathways but also closely linked to genome plasticity driven by CIN [30]. In the high‐risk group, CIN induced by TP53 mutations enhances tumor cell sensitivity to drugs such as 5‐fluorouracil and paclitaxel, which act by interfering with DNA synthesis and mitosis; cells with genomic instability are more vulnerable to such disruptions [31]. In contrast, the low‐risk group characterized by CTNNB1‐ARID2 comutations may alter cellular response patterns to drugs like gemcitabine and cytarabine by regulating chromatin remodeling and DNA repair‐related pathways. These pathway‐level differences cannot be explained solely by single drug targets but involve multidimensional adaptive changes in tumor cells, including metabolic reprogramming and stress responses [32]. The comutation frequency of CTNNB1 and ARID2 is approximately 5%, significantly higher than random co‐occurrence. CTNNB1 activates Wnt/*β*‐catenin signaling and suppresses expression of SWI/SNF core subunits, whereas ARID2 loss directly impairs chromatin accessibility of DNA repair genes by the PBAF complex, resulting in defects in homologous recombination and nucleotide excision repair. Functional studies show that ARID2‐deficient cells exhibit a twofold–threefold decrease in IC50 for gemcitabine and cisplatin, and this sensitization effect can be reversed by ARID2 complementation [33]. Moreover, CTNNB1 activation itself downregulates ATM/ATR signaling and increases replication stress, further sensitizing HCC cells to nucleoside analogs such as gemcitabine and cytarabine [34]. Therefore, this comutation remodels tumor cell response patterns to nucleoside analogs via a dual mechanism of “chromatin remodeling impairment + amplification of replication stress,” providing a potential biomarker for precision chemotherapy stratification.

Notably, in the drug sensitivity prediction analysis of this study, the IC50 values of gemcitabine and cytarabine exhibited inconsistent variation trends with the RiskScore (Figure 8e,f), contrary to the model′s expected logic that high‐RiskScore patients would exhibit higher sensitivity (lower IC50 values). This indicates the model cannot effectively distinguish the sensitivity to these two drugs. This phenomenon may be attributed to their mechanism of action: both gemcitabine and cytarabine are deoxycytidine analogs, and their cytotoxicity primarily relies on nucleoside transporter (hENT1 and CNT1)‐mediated membrane uptake and subsequent phosphorylation by deoxycytidine kinase (DCK), rather than directly targeting DNA double‐strand breaks or chromosome segregation processes [35]. Thus, even in high‐CIN cells with a background of persistent DNA damage, insufficient nucleoside transport/phosphorylation efficiency hinders the amplification of the “replicative stress” effect induced by these drugs. Furthermore, in vitro drug sensitivity data are derived from cell lines, which may underestimate the impact of rapid clearance by hepatic enzymes in vivo, further exacerbating the inconsistency between CIN characteristics and IC50 variation trends [36]. In summary, the current model has limited predictive value for nucleoside analogs, and future evaluations should integrate the expression of drug‐metabolizing enzymes or transporter activity for a comprehensive assessment.

In targeted therapy, characteristic mutations in high‐risk groups, such as TP53 and RB1, are linked to aberrant signaling pathways, providing broad opportunities for target selection [27]. Activation of pathways like PI3K/AKT and MAPK has become anchor points for the design of various targeted drugs [37, 38]. However, drug resistance remains a significant clinical challenge, with tumor heterogeneity as a key contributing factor. The molecular features corresponding to the risk stratification revealed in this study provide potential biomarkers for predicting resistance risk. Moreover, with the advancement of precision medicine, in addition to multikinase inhibitors such as sorafenib, drugs targeting single molecules like VEGF and c‐MET have emerged continuously [39]. The advantages of lenvatinib in inhibiting angiogenesis and the specificity of cabozantinib against aberrant c‐MET activation offer hope for HCC patients with diverse molecular phenotypes [40]. Nevertheless, overcoming resistance through combination therapies, such as combining targeted agents with ICIs, remains an urgent problem to solve. This requires a deeper understanding of how targeted therapies remodel the tumor immune microenvironment and optimization of treatment modalities regarding timing and dosing [41]. The risk model constructed in this study, if further integrated with multiomics features including immune and metabolic profiles, may provide stronger support for the precise design of such combination regimens.

Clinical staging is a core basis for prognosis assessment and treatment decision‐making in HCC patients. In this study, staging was significantly associated with risk scores, TMB, and other molecular features. The high‐risk group was predominantly in advanced stages, accompanied by enhanced CIN and an immunosuppressive microenvironment. By integrating clinical staging with molecular indicators in the constructed nomogram model, predictive accuracy was significantly improved.

From the treatment perspective, differences in the immune microenvironment provide a basis for precise selection of therapeutic strategies. Previous studies have shown that the high‐risk group, characterized by a dominant immunosuppressive network, exhibits limited response to immune checkpoint inhibitor monotherapy. However, chemotherapy can induce immunogenic cell death of tumor cells and remodel the immune microenvironment, thereby synergizing with immunotherapy [42]. Targeted therapies can inhibit pathways such as VEGF, improving abnormal tumor vasculature and alleviating immunosuppression, which in turn enhances the efficacy of immunotherapy [43]. The low‐risk group′s relatively favorable immune microenvironment suggests they are more likely to benefit from immunotherapy. Importantly, our risk model incorporates diverse datasets—such as genomics and transcriptomics—to accurately characterize immune microenvironment features and their association with treatment response.

In this study, functional cellular experiments were conducted to deeply validate the biological roles of core genes SSRP1 and SETDB1 in HCC. siRNA‐mediated gene knockdown significantly inhibited HCC cell proliferation and invasion, clearly demonstrating that these two genes play critical roles in maintaining the malignant phenotype of tumor cells. These findings not only functionally support the important prognostic impact of the core genes identified in the risk‐scoring model but also provide experimental evidence for exploring their molecular mechanisms. Specifically, SSRP1, as a histone‐binding protein, may promote tumor cell proliferation by regulating chromatin structure and gene transcriptional activity, whereas SETDB1, a histone methyltransferase involved in epigenetic modification, may facilitate tumor cell invasion and metastasis. These cellular‐level discoveries enrich our understanding of chromosome instability‐associated genes in HCC progression and reveal potential therapeutic targets. In the future, targeted intervention strategies against SSRP1 and SETDB1 might effectively suppress tumor growth and metastasis, thereby improving clinical outcomes for patients, warranting further mechanistic studies and drug development.

The present investigation concentrated on HCC, leveraging chromosome instability‐related genes as the foundational basis. Crucial gene modules were identified through WGCNA, followed by the selection of the most effective predictive model from a pool of 101 machine learning algorithms. By synthesizing multiomics data, a robust and precise risk scoring system was developed. This prognostic model not only enables accurate stratification of patient outcomes and delineates pronounced disparities in molecular features and the tumor immune microenvironment between high‐ and low‐risk groups but also facilitates individualized therapeutic decision‐making for chemotherapy and immunotherapy. Specifically, high‐risk patients demonstrate increased sensitivity to chemotherapeutic agents such as 5‐fluorouracil and paclitaxel, making them suitable candidates for intensified chemotherapy regimens. Conversely, patients classified as low‐risk tend to respond better to targeted treatments or less intensive chemotherapy. Moreover, the high‐risk group is characterized by elevated expression of immune checkpoint molecules (e.g., PDCD1, CD276, and CD47) and higher TIDE scores, indicating that monotherapy with ICIs may have limited effectiveness, whereas combination immunotherapy or innovative immunoregulatory approaches could offer improved outcomes. Validated through multifactor algorithms and large sample cohorts, the model demonstrates excellent stability and reliability, providing a solid foundation for clinical individualized treatment decisions and exhibiting considerable potential for clinical translation and application.

Nevertheless, this study has several limitations. First, the model was primarily constructed based on retrospective data from public databases, with a limited sample size for internal validation, which may introduce selection bias and affect the model′s generalizability in real‐world clinical settings. Second, the data were derived from a single center, lacking clinical diversity across multiple regions and institutions, thereby raising uncertainty regarding the model′s applicability to patients from different clinical backgrounds. Finally, prospective studies or validations based on real‐world external cohorts have not yet been conducted, making it difficult to comprehensively assess the model′s predictive accuracy and utility in dynamic clinical practice, thereby limiting its broader clinical implementation.

## 5. Conclusion

In summary, the CIN‐associated gene‐based HCC risk‐scoring model constructed in this study overcomes the single‐dimensional limitations of traditional prognostic models and achieves dual guidance for patient prognosis and therapeutic strategies, demonstrating significant clinical application potential. Future efforts should incorporate multicenter, large‐sample prospective studies to further validate and optimize the model, ensuring its stability and wide applicability in clinical practice, and promoting the continued advancement of precision diagnosis and treatment for HCC.

## Author Contributions

Zehao Li and Xiao Hu: writing—original draft, formal analysis, conceptualization. Boqiang Zhong: writing—review and editing, supervision. Qian Zhang: supervision. Lin Sun: writing—review and editing, supervision. Xiaoxiao Li: conceptualization.

## Funding

This study was supported by National Natural Science Foundation of China (10.13039/501100001809, 52075277); Medical and Health Scientific Research Project of Qingdao (2024‐WJKY164); Qingdao Outstanding Health Professional Development Fund and Wu Jieping Medical Foundation Youth Clinical Research Special Project—Digestive and Urinary Tumor Special Project (320.6750.2025‐02‐9); “Clinical Research Project on Digestive Tract Tumors” of China International Medical Exchange Foundation (Z‐2014‐08‐2503).

## Conflicts of Interest

The author declares no conflicts of interest.

## Supporting information


**Supporting Information** Additional supporting information can be found online in the Supporting Information section. Table S1 A list of 101 machine learning models and algorithm combinations used for prognostic gene screening and risk model construction in hepatocellular carcinoma, including basic algorithms such as Lasso, CoxBoost, StepCox, random survival forest (RSF), and integrated models with parameter combinations like different alpha values of elastic net (Enet). Table S2: A list of 32 core genes associated with chromosomal instability (CIN) identified based on the study by Drews RM et al., including key genes such as AKT1, BRCA1, and TP53. Table S3: A table of quantitative chromosomal instability (CIN) scores of hepatocellular carcinoma samples in the TCGA dataset calculated by Gene Set Variation Analysis (GSVA) algorithm, including the CIN score corresponding to each TCGA sample ID. Table S4: A table of information on the Top 200 core genes highly correlated with CIN screened by weighted gene coexpression network analysis (WGCNA) in the TCGA dataset, including gene probe names, colors of associated coexpression modules, correlation coefficients with CIN, and correlation *p* values. Table S5: A table of univariate Cox regression analysis results of CIN‐related genes in the TCGA and GSE54236 datasets, including gene ID, hazard ratio (HR), 95% confidence interval of HR, and prognostic correlation *p* value. Table S6: A list of candidate prognostic genes associated with CIN shared by the TCGA and GSE54236 datasets obtained through intersection analysis, totaling 73 genes. Table S7: A comprehensive dataset integrating clinical information of samples in the TCGA dataset and risk prediction results of 101 machine learning models, including sample ID, overall survival time (OS.time), overall survival status (OS), cohort affiliation, and the risk prediction value of each model for the sample. Table S8: A list of 20 core prognostic genes associated with CIN screened by the optimal models of StepCox [both] and CoxBoost.

## Data Availability

The original contributions presented in the study are included in the article/Supporting Information. Further inquiries can be directed to the corresponding authors.
